# Boosting Antitumor Sonodynamic Therapy Efficacy of Black Phosphorus via Covalent Functionalization

**DOI:** 10.1002/advs.202102422

**Published:** 2021-08-13

**Authors:** Yajuan Liu, Zhiyuan Li, Feng Fan, Xianjun Zhu, Lingbo Jia, Muqing Chen, Pingwu Du, Lihua Yang, Shangfeng Yang

**Affiliations:** ^1^ Hefei National Laboratory for Physical Sciences at Microscale CAS Key Laboratory of Materials for Energy Conversion Department of Materials Science and Engineering Anhui Laboratory of Advanced Photon Science and Technology University of Science and Technology of China Hefei 230026 China; ^2^ Hefei National Laboratory for Physical Sciences at Microscale CAS Key Laboratory of Soft Matter Chemistry School of Chemistry and Materials Science University of Science and Technology of China Hefei 230026 China; ^3^ College of Electronic and Optical Engineering and College of Microelectronics Nanjing University of Posts and Telecommunications Nanjing 210023 China

**Keywords:** black phosphorus, covalent functionalization, fullerene, reactive oxygen species, sonodynamic therapy

## Abstract

Sonodynamic therapy (SDT) triggered by ultrasound represents an emerging tumor therapy approach with minimally invasive treatment featuring nontoxicity and deep tissue‐penetration, and its efficacy sensitively depends on the sonosensitizer which determines the generation of reactive oxygen species (ROS). Herein, for the first time covalently functionalized few‐layer black phosphorus nanosheets (BPNSs) are applied as novel sonosensitizers in SDT, achieving not only boosted SDT efficacy but also inhibited cytotoxicity relative to the pristine BPNSs. Three different covalently functionalized‐BPNSs are synthesized, including the first fullerene‐functionalized BPNSs with C_60_ covalently bonded onto the surface of BPNSs (abbreviated as C_60_‐s‐BP), surface‐functionalized BPNSs by benzoic acid (abbreviated as BA‐s‐BP), and edge‐functionalized BPNSs by C_60_ (abbreviated as C_60_‐e‐BP), and the role of covalent functionalization pattern of BPNSs on its SDT efficacy is systematically investigated. Except C_60_‐e‐BP, both surface‐functionalized BPNSs (C_60_‐s‐BP, BA‐s‐BP) exhibit higher SDT efficacies than the pristine BPNSs, while the highest SDT efficacy is achieved for BA‐s‐BP due to its strongest capability of generating the hydroxyl (·OH) radicals, which act as the dominant ROS to kill the tumor cells.

## Graphical abstract

Covalently functionalized few‐layer black phosphorus nanosheets (BPNSs), including surface‐functionalized BPNSs by benzoic acid (BA‐s‐BP) or C_60_ (C_60_‐s‐BP) and edge‐functionalized BPNSs by C_60_ (C_60_‐e‐BP), are applied as novel sonosensitizers in sonodynamic thearpy (SDT) for the first time, resulting in boosted SDT efficacy of BPNSs. BA‐s‐BP generates the highest amount of the hydroxyl (·OH) radicals to kill the tumor cells.

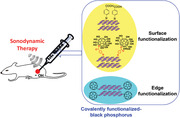

## 1 Introduction

Anticancer therapy has drawn worldwide attention since cancer has become the world's biggest killer. Versatile dynamic therapies, including photodynamic,^[^ ^1^, ^2^ ^]^ sonodynamic,^[^ ^3^ ^]^ radiodynamic,^[^ ^4^ ^]^ chemodynamic,^[^ ^5^, ^6^ ^]^ and electrodynamic therapies,^[^ ^7^ ^]^ have been widely used in tumor treatment via generating reactive oxygen species (ROS) to kill tumor cells under stimulating sources. Compared with traditional chemotherapy, dynamic therapy avoids drug resistance through generating ROS to oxidize cellular DNA, protein, lipid.^[^ ^1^ ^]^ Noteworthy, external stimuli are usually needed in dynamic therapy. For example, photodynamic therapy (PDT) has been commonly implemented in clinic as a noninvasive tumor therapy technique, but it requires light irradiation and suffers from low tissue‐penetrating depth of light.^[^ ^1^, ^2^ ^]^ Alternatively, sonodynamic therapy (SDT) triggered by ultrasound represents an emerging tumor therapy approach with minimally invasive treatment.^[^ ^8^, ^9^, ^10^, ^11^ ^]^ Ultrasound can activate the sonosensitizer to generate ROS, which kill the cancer cells via apoptosis and necrosis.^[^ ^11^ ^]^ Since ultrasound can be focused deeply within tissues and penetrate up to several tens of centimeters, the critical limitation of PDT in low tissue‐penetrating depth can be overcome.^[^ ^8^ ^]^ Such an advantage enables SDT as a promising noninvasive tumor therapeutic approach, and its efficacy sensitively depends on the sonosensitizer which determines the generation of ROS. Two major types of sonosensitizers have been reported, including organic (such as porphyrin derivatives,^[^ ^12^ ^]^ xanthene dyes^[^ ^13^, ^14^ ^]^) and inorganic (such as TiO_2_,^[^ ^15^ ^]^ MnWO*_X_* nanoparticles,^[^ ^16^ ^]^ single‐layer WS_2_,^[^ ^17^ ^]^ SiO_2_ nanoparticles, silicon nanoparticles^[^ ^8^ ^]^) sonosensitizers. Among them, organic sonosensitizers generally have relatively low stability under ultrasound exposure, and exhibit phototoxicities and skin sensitivity after treatment.^[^ ^16^ ^]^ For the conventional inorganic sonosensitizers such as TiO_2_ nanoparticles, despite its high chemical stability and low phototoxicity, the low quantum yield of ROS generation originated from rapid charge carrier combination limits their SDT efficacy.^[^ ^16^, ^18^ ^]^ Besides, the low degradability of inorganic compounds would be detrimental for in vivo applications.^[^ ^15^, ^16^ ^]^ Therefore, it is urgently desired to develop novel sonosensitizers with not only high sonosensitizing efficiency but also considerable biocompatibility and safety.

Elemental 2D materials consisting of atomically thin sheets such as graphene and black phosphorus (BP) have been attracting great attention in the past decade, showing considerable biocompatibility and thus promising biomedical applications.^[^ ^19^, ^20^, ^21^, ^22^, ^23^, ^24^, ^25^, ^26^, ^27^, ^28^, ^29^ ^]^ In particular, BP exhibits extraordinary band structure featuring thickness‐dependent tunable direct bandgap of ≈0.3–2.0 eV and high charge carrier mobility of ≈1000 cm^2^ V^−1^ s^−1^, enabling its potential applications in transistors, energy conversion, and storage, catalysis, and biomedicine, etc.^[^ ^24^, ^25^, ^26^, ^27^, ^28^, ^29^ ^]^ Given that the optical absorption of BP is tunable, across a broad range from UV to visible owing to its thickness‐dependent bandgap, few‐layer BP nanosheets (BPNSs) have been applied as effective photosensitizers in PDT since the discovery of their ability in efficient generation of singlet oxygen (^1^O_2_) radicals in 2015 (ref. [30]). Because many sonosensitizers applicable in SDT are derived from photosensitizers,^[^ ^16^ ^]^ this stimulates the feasibility of applying BPNSs in SDT. Indeed, very recently we demonstrated the first application of BPNSs as novel sonosensitizer of SDT, which shows ultrasound‐excited cytotoxicity to cancer cells via generation of hydroxyl (·OH) radicals like ROS.^[^ ^31^ ^]^ However, such a seminal work is based on the pristine BPNSs, which are known to have low ambient stability, thus limiting their SDT applications undoubtedly. It has been revealed that, due to the existence of a pair of lone electrons in each phosphorus atom of the puckered layer, BPNSs can readily react with oxygen and water adsorbed on their surface, resulting in the low ambient stability of BPNSs.^[^ ^32^, ^33^, ^34^, ^35^, ^36^, ^37^, ^38^, ^39^ ^]^ Therefore, improving the ambient stability of BPNSs is a prerequisite for their biomedical applications. On the other hand, the toxicity of BPNSs is another critical concern for their practical applications.^[^ ^40^ ^]^ Very recently it was revealed that ROS generated during the degradation of BPNSs was the source of its cytotoxicity, which is dependent on the dose.^[^ ^40^ ^]^ While BPNSs can effectively kill tumor cells at low doses, they still exhibit cytotoxicity to normal cells at high concentrations.^[^ ^41^ ^]^ Hence, it is challenging to boost the SDT efficacy of BPNSs and simultaneously inhibit their cytotoxicity.

Herein, we rationally design and synthesize the first fullerene‐functionalized BPNSs with C_60_ covalently bonded onto the surface of BPNSs (abbreviated as C_60_‐s‐BP). Combining two other functionalized BPNSs including surface‐functionalized BPNSs by benzoic acid (abbreviated as BA‐s‐BP) and edge‐functionalized BPNSs by C_60_ (abbreviated as C_60_‐e‐BP), we systematically investigate the role of covalent functionalization pattern on the SDT efficacy and cytotoxicity of BPNSs. Except C_60_‐e‐BP, both surface‐functionalized BPNSs (C_60_‐s‐BP, BA‐s‐BP) exhibit higher SDT efficacies than the pristine BPNSs, while the highest SDT efficacy is achieved for BA‐s‐BP. These results are interpreted by their difference on the capability of generation of the hydroxyl (·OH) radicals, which act as the dominant ROS to kill the tumor cells. Our study demonstrates for the first time that covalent functionalization is an effective approach to boost the SDT efficacy of BP, opening up a new avenue for biomedical applications of BP.

## 2 Results and Discussion

Since the seminal work on covalently functionalizing BPNSs via aryl diazonium reaction by Hersam et al. in 2016 (ref. [42]), covalent functionalizations have been developed as one of the most effective approaches in passivating the phosphorus atom bearing lone electron pair and consequently improving the ambient stability of BPNSs.^[^ ^34^, ^35^, ^36^, ^37^, ^38^, ^39^ ^]^ Very recently, we fulfilled covalent functionalizations of BPNSs in both surface‐ and edge‐selective addition patterns, including surface‐functionalization by azabenzoic acid (abbreviated as BA‐s‐BP)^[^ ^34^ ^]^ and edge‐functionalization by fullerene C_60_ (abbreviated as C_60_‐e‐BP) via a solid‐state mechanochemical method,^[^ ^37^ ^]^ and succeeded in improving the ambient stability of BPNSs. Particularly, fullerene has high ambient stability against oxygen and water,^[^ ^43^, ^44^ ^]^ and versatile biomedical applications of fullerenes have been reported on the basis of their good biocompatibility.^[^ ^45^, ^46^, ^47^ ^]^ However, covalently bonding C_60_ molecules onto the surface of BPNSs has never been reported, which is more preferable than edge‐functionalization since passivation of the phosphorus atoms on the surface of BPNSs with much larger fraction than edge atoms is expected to result in better passivation effect. Hence, in our present work we are stimulated to prepare the first fullerene‐functionalized BPNSs with C_60_ covalently bonded onto the surface of BPNSs via a solution‐based approach.

**Figure 1:**
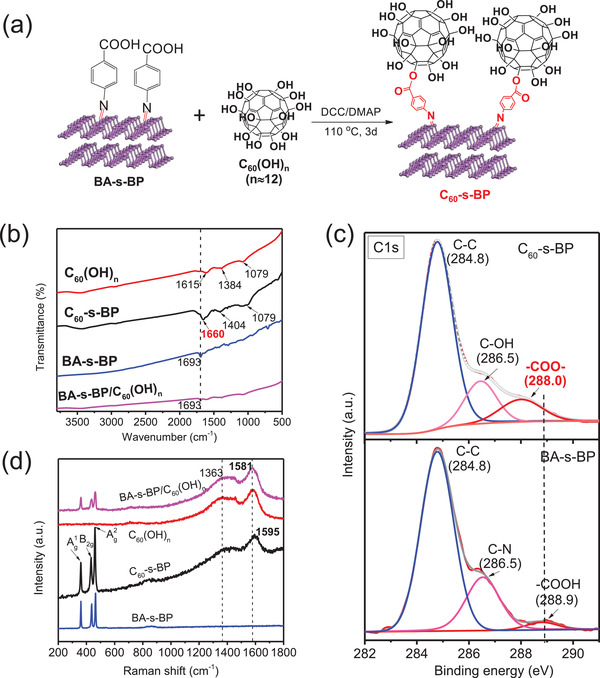
a) Scheme of the preparation process and structure of C_60_‐s‐BP. b) FT‐IR spectra of C_60_(OH)*_n_*, BA‐s‐BP, C_60_‐s‐BP, C_60_(OH)*_n_*/BA‐s‐BP. c) High‐resolution C1s XPS spectra of C_60_‐s‐BP and pristine BA‐s‐BP. d) Raman spectra of C_60_‐s‐BP, BA‐s‐BP/C_60_(OH)*_n_*, C_60_(OH)*_n_*, and BA‐s‐BP.

Novel fullerene‐functionalized BPNSs with C_60_ covalently bonded onto the surface of BPNSs (abbreviated as C_60_‐s‐BP) is synthesized via a facile esterification reaction between the benzoic acid surface‐functionalized BPNSs (BA‐s‐BP) and fullerenol (**Figure** **1**a). BA‐s‐BP was synthesized by stirring a mixture of BPNSs and 4‐azidobenzoic acid in dimethyl formamide (DMF) as we reported previously (see also Figure S1, Supporting Information), featuring covalently grafting of azabenzoic acid dominantly onto the surface of BPNSs via P═N double bonds despite of the existence of a small amount of azabenzoic acid moiety at the edge of BPNSs.^[^ ^34^ ^]^ Grafting of the carboxyl groups (—COOH) in BA‐s‐BP renders a possibility of post‐functionalization, such as esterification reaction with the hydroxyl (—OH) groups. In specific, hydroxylated fullerene (fullerenol, C_60_(OH)*_n_*) not only contains the hydroxyl groups but also shows high water solubility and good biocompatibility,^[^ ^45^, ^46^, ^47^ ^]^ thus is selected as the ideal reactant for esterification with BA‐s‐BP. C_60_(OH)*_n_* with an estimated average n value of 12 (see Figure S2 and Table S1, Supporting Information) was prepared by stirring C_60_ in toluene mixed with an oxygenated aqueous NaOH solution using tetrabutylammonium hydroxide as a phase transfer catalyst as reported in literatures.^[^ ^45^ ^]^ Then, 5 mg BA‐s‐BP dispersed in DMF was mixed with 10 mg C_60_(OH)*_n_* in an ambient atmosphere, followed by adding 111 mg dicyclohexylcarbodiimide (DCC) and 24 mg 4‐dimethylaminopyridine (DMAP) as catalyst. After heating the mixture solution at 110 °C for 3 days with intense stirring, brown suspension was obtained, which was centrifuged at 12 000 rpm for 30 min and washed with DMF for three times to remove the raw materials and dried in a vacuum oven at 50 °C for 48 h (see Experimental Section for details).

A series of spectroscopic characterizations were carried out to confirm the successful esterification reaction affording covalent bonding of C_60_ molecules onto the surface of BPNSs within C_60_‐s‐BP. The transformation of the carboxyl groups (—COOH) in BA‐s‐BP to the ester groups (—COO—) in C_60_‐s‐BP was first verified by Fourier‐transform infrared (FT‐IR) spectroscopy. Figure 1b compares the FT‐IR spectra of BA‐s‐BP, C_60_(OH)*_n_*, and C_60_‐s‐BP, which includes also that of a physical mixture of BA‐s‐BP and C_60_(OH)*_n_* (abbreviated as BA‐s‐BP/C_60_(OH)*_n_*, in which the weight ratio of C_60_(OH)*_n_* is ≈28%, close to that within C_60_‐s‐BP as discussed below). Clearly, the characteristic vibrational peak of the carboxyl groups (‐COOH) at 1693 cm^−1^ detected in both BA‐s‐BP^[^ ^34^ ^]^ and BA‐s‐BP/C_60_(OH)*_n_* shifts negatively to 1660 cm^−1^, which is assigned to the ester groups (—COO—) in C_60_‐s‐BP.^[^ ^48^ ^]^ Besides, the four characteristic vibrational peaks at 3442, 1615, 1384, and 1079 cm^−1^ of C_60_(OH)*_n_*, corresponding to the —OH groups, C═C bonds, O—H bending, and C—O stretching modes in C—OH, respectively,^[^ ^49^ ^]^ are observed in the spectrum of C_60_‐s‐BP with negligible shifts. It is worth noting that the signal of O—H bending mode exhibits an apparent blue‐shift from 1384 cm^−1^ for C_60_(OH)*_n_* to 1404 cm^−1^ for C_60_‐s‐BP, suggesting the change of the chemical environment of the —OH groups as a result of the ester group (—COO—) formation. These results indicate the occurrence of esterification reaction between BA‐s‐BP and C_60_(OH)*_n_*, affording successful grafting of C_60_ moiety within C_60_‐s‐BP instead of a physical mixture.

The formation of the ester groups (—COO—) in C_60_‐s‐BP is further proven by X‐ray photoemission spectroscopic (XPS) measurements. According to the XPS survey spectrum of BA‐s‐BP, apparently the signal intensity of carbon atoms has increased (see Figure S4, Supporting Information). The atomic ratio of C and P increased from 1.51 for BA‐s‐BP to 6.13 for C_60_‐s‐BP, consistent with the grafting of C_60_. Figure 1c compares the high‐resolution C1s XPS spectra of BA‐s‐BP and C_60_‐s‐BP. In the C1s XPS spectrum of BA‐s‐BP, two intense peaks centered at 284.8 and 286.5 eV correspond to C═C and C—N bonds respectively, and a broad shoulder peak at 288.9 eV is assigned to the —COOH group.^[^ ^50^, ^51^ ^]^ After C_60_ bonding, the latter peak negatively shifts to 288.0 eV, which is attributed to —COO— group.^[^ ^48^, ^50^ ^]^ In addition, C_60_ bonding imposes negligible perturbation on the BA‐s‐BP moiety according to the comparison of the high‐resolution P2p XPS spectra of BA‐s‐BP and C_60_‐s‐BP (see Figure S5, Supporting Information).

We then carried out Raman spectroscopic characterization to probe the existence of C_60_ moiety within C_60_‐s‐BP. Figure 1d compares Raman spectra of BA‐s‐BP, C_60_(OH)*_n_*, C_60_‐s‐BP, and BA‐s‐BP/C_60_(OH)*_n_*. BA‐s‐BP exhibit three intense Raman signals at 361, 437, and 465 cm^−1^ assigned respectively to $A_{g}^{1}$, B_2g_, and $A_{g}^{2}$ modes, which are almost identical to the pristine BPNSs.^[^ ^34^ ^]^ Raman spectrum of BA‐s‐BP/C_60_(OH)*_n_* mixture is simply a superposition of signals of BA‐s‐BP and C_60_(OH)*_n_* without any shifts, indicating negligible intermolecular interactions between BA‐s‐BP and C_60_(OH)*_n_* moieties. However, for C_60_‐s‐BP, its Raman spectrum is apparently different from that of BA‐s‐BP/C_60_(OH)*_n_* especially the up‐shift (≈14 cm^−1^) of the most intense characteristic peak of C_60_(OH)*_n_* at 1581 cm^−1^ assigned to carbon cage vibration. Such an up‐shift suggests strong intermolecular interactions between BA‐s‐BP and C_60_(OH)*_n_* moieties. On the other hand, the three characteristic Raman peaks of BA‐s‐BP show negligible shifts, instead the relative intensity of $A_{g}^{1}$/$A_{g}^{2}$ decreases in the spectrum of C60‐s‐BP, consistent with the change of surface structures of BA‐s‐BP. All these results confirm that C_60_‐s‐BP is not a physical mixture of BA‐s‐BP and C_60_(OH)*_n_* but a covalent hybrid. According to the elemental analysis of C_60_‐s‐BP, the molar ratio of C_60_ moiety is ≈1.13%, corresponding to an average C_60_ molar content of 11.3 per 1000 phosphorus atoms (see Table S2, Supporting Information).

**Figure 2:**
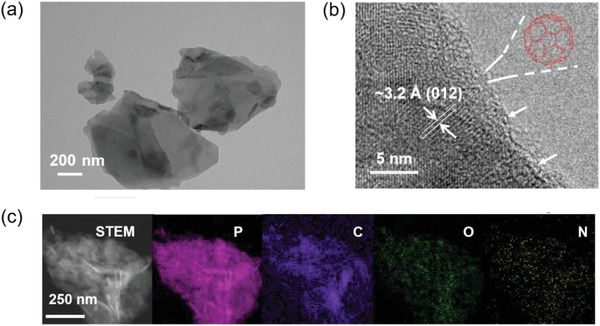
a) TEM image of C_60_‐s‐BP. b) HR‐TEM image of C_60_‐s‐BP. The arrows mark the C_60_ molecules. Note that the majority of C_60_ molecules grafted on the surface of BPNSs are invisible due to limited resolution of our instrument. c) STEM and EDX elemental (P, C, O, and N) mapping images of C_60_‐s‐BP.

It is intriguing to investigate the influence of C_60_ bonding on the morphology, crystallinity, and optical absorption properties of BPNSs. We first carried out scanning electron microscopic (SEM) study to investigate the morphology of C_60_‐s‐BP. While the pristine BPNSs show large sheets with sizes of more than 1 µm, after azide functionalization BA‐s‐BP exhibits smaller nanosheets with size of 400 nm in average, which is comparable to that of C_60_‐s‐BP (see Figure S7, Supporting Information). This indicates that C_60_ bonding has negligible influence on the 2D‐layered structure of BA‐s‐BP.^[^ ^34^ ^]^ SEM image of the pristine C_60_(OH)*_n_* shows irregular aggregated particles with sizes of more than 1 µm, which are however not observed in the image of C_60_‐s‐BP, indicating that C_60_ molecules distribute separately on the surface of BPNSs. This phenomenon is consistent with the transmission electron microscopic (TEM) results. TEM images of BA‐s‐BP and C_60_‐s‐BP both show flat nanosheets, and the average size of an individual sheet of C_60_‐s‐BP is comparable to that of the pristine BA‐s‐BP (**Figure** **2**a and Figure S8a, Supporting Information). Again, the aggregated particles observed in the TEM image of pristine C_60_(OH)*_n_* (see Figure S8b, Supporting Information) are invisible in the image of C_60_‐s‐BP. On the other hand, according to atomic force microscopic (AFM) measurements, the average thickness of C_60_‐s‐BP is 5.40 nm, which is smaller than that of BA‐s‐BP (10.84 nm), indicating that C_60_ functionalization promotes the exfoliation of BPNSs (Figure S9, Supporting Information).

In the high‐resolution TEM (HR‐TEM) image of C_60_‐s‐BP (Figure 2b), the discernible lattice fringes of (012) plane with space distance of ≈3.2 Å are observed, whereas for the pristine BA‐s‐BP the detected lattice fringes are correlated to (111) plane with a space distance of ≈2.5 Å instead (Figure S10, Supporting Information).^[^ ^34^ ^]^ This reveals that C_60_ bonding dramatically affects the crystallinity of BA‐s‐BP, as confirmed further by X‐ray diffraction (XRD) analyses. According to a comparison of the XRD patterns of BA‐s‐BP, C_60_(OH)*_n_* and C_60_‐s‐BP (Figure S11, Supporting Information), the characteristic diffraction peaks of BA‐s‐BP at 34.2° and 52.3°, which are indexed as the (040) and (060) planes of BP,^[^ ^34^ ^]^ are clearly detected in the XRD pattern of C_60_‐s‐BP, whereas the intensive diffraction peak of BA‐s‐BP at 16.8° corresponding to (020) plane disappears in the XRD pattern of C_60_‐s‐BP. Instead, two new peaks at 27.6°and 36.0°appear, which are indexed respectively as the (021) and (111) planes of BP typically available in functionalized BP.^[^ ^52^ ^]^ No diffraction signal of C_60_ is observed since C_60_(OH)*_n_* exhibits amorphous feature.^[^ ^45^, ^46^ ^]^ Moreover, the HR‐TEM image of C_60_‐s‐BP looks more disordered than that of the pristine BA‐s‐BP, with the lattice fringes being less discernible. This is consistent with the decreased crystallinity of BPNSs within C_60_‐s‐BP. Interestingly, in the HR‐TEM image of the C_60_‐s‐BP, hollow spherical species with diameter of ≈1.0 nm are observed (Figure 2b), which are however absent in the image of the pristine BA‐s‐BP (Figure S10, Supporting Information). The diameter of such hollow spherical species is close to the van der Walls diameter of C_60_ molecules (≈1.0 nm^[^ ^37^ ^]^), confirming once more the successful bonding of C_60_. The elemental distributions of C_60_‐s‐BP are further probed by scanning transmittance electron microscopy‐energy dispersive X‐ray spectroscopy (STEM‐EDX), revealing that the phosphorus (P), carbon (C), oxygen (O), and nitrogen (N) elements all distribute over the entire nanosheets homogeneously (Figure 2c).

It has been extensively reported that BPNSs are very reactive to oxygen and water under ambient conditions due to the existence of lone electron pair in each phosphorus atom, resulting in poor ambient stability of BPNSs.^[^ ^32^, ^33^, ^34^, ^35^, ^36^, ^37^, ^38^, ^39^ ^]^ Thus, it is intriguing to check whether covalently bonding the stable C_60_ molecules onto the surface of BPNSs may improve their ambient stability or not. Given that oxidation of BPNSs in the presence of water leads to the formation of phosphoric acid and consequently degrading the semiconducting nature of BPNSs, UV/Vis optical absorption property can be used as a simple metric of the degradation degree of BPNSs.^[^ ^33^, ^34^, ^35^, ^36^, ^37^, ^38^, ^39^ ^]^ We monitored the optical absorbances of C_60_‐s‐BP and the pristine BPNSs which are both dispersed in water (without deoxygenation) and stored under ambient condition for 21 days. While the UV/Vis absorbance of the pristine BPNSs dispersion at 460 nm degrades gradually with the degradation ratio reaching 48% after standing for 21 days (Figure S12a, Supporting Information), after C_60_ functionalization, the degradation ratio of C_60_‐s‐BP after standing for 21 days decreases dramatically to ≈14% only (Figure S12b, Supporting Information). This reveals that C_60_ functionalization can improve the ambient stability of BPNSs with the degradation ratio inhibited by 3.4 times (Figures S12c,d, Supporting Information). Noteworthy, for the reported C_60_ edge‐functionalized BPNSs (C_60_‐e‐BP), despite of the improvement of the ambient stability relative to the pristine BPNSs, the degradation ratio of its aqueous dispersion after standing for 7 days is 14%,^[^ ^37^ ^]^ which is ≈eleven times higher than that for C_60_‐s‐BP (≈1.3%, see Figure S12d, Supporting Information). This indicates that C_60_ bonding onto the surface of BPNSs within C_60_‐s‐BP is more efficient in protecting BPNSs from attacks of oxygen and water than the case of C_60_ bonding at the edge of BPNSs.

**Figure 3:**
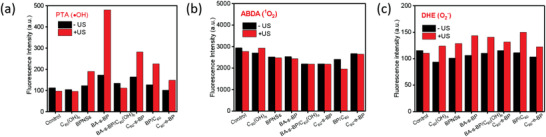
a) Fluorescence emission intensity of PTA specifically used to probe ·OH radicals. b) Fluorescence emission intensity of ABDA specifically used to probe ^1^O_2_ radicals. c) Fluorescence emission intensity of DHE specifically used to probe O_2_^−^ radicals. −US refers to the presence of 50 µg mL^−1^ of BPNSs in PBS solution without ultrasound irradiation, and +US refers to ultrasound excitation at the output power density of 1.0 W cm^−2^, 1.0 MHz.

Given that C_60_‐s‐BP, C_60_‐e‐BP, and BA‐s‐BP based on different covalent functionalization patterns (surface versus edge) and addend groups can all improve the ambient stability of BPNSs, next we managed to apply them as novel sonosensitizers for SDT and to systematically investigate the role of covalent functionalization pattern of BPNSs on their SDT efficacy. We first evaluated their capabilities of ROS generation upon ultrasound activation. It has been reported that the pristine BPNSs can generate the hydroxyl (·OH) radicals as the dominant ROS, whose yield is promoted under ultrasound exposure.^[^ ^31^ ^]^ We first used p‐phthalic acid (PTA) as a fluorescent probe, which is exclusively sensitive to ·OH radicals,^[^ ^31^ ^]^ to detect the ·OH radical generation capabilities of C_60_‐s‐BP, C_60_‐e‐BP, and BA‐s‐BP according to the fluorescence intensity. For comparison, three control samples, including the pristine C_60_(OH)*_n_* and BPNSs, BA‐s‐BP/C_60_(OH)*_n_* and a physical mixture of BPNSs and C_60_ (abbreviated as BP/C_60_), were also detected. As shown in **Figure** **3**a, under ultrasound stimulation (1.0 W cm^−2^, 1.0 MHz), the fluorescence intensity of PTA at ≈425 nm increases dramatically for C_60_‐s‐BP and BA‐s‐BP as well as BP/C_60_, whereas the pristine BPNSs and C_60_‐e‐BP both exhibit smaller increases on the fluorescence intensity increase. Among them, BA‐s‐BP delivers the highest fluorescence intensity, suggesting that BA‐s‐BP generates the highest amount of ·OH radicals, which is increased by ≈2.8 times relative to that obtained without ultrasound. Upon grafting further C_60_ molecules onto BA‐s‐BP, the as‐prepared C_60_‐s‐BP shows decreased fluorescence intensity relative to BA‐s‐BP due likely to the radical scavenging property of C_60_ (ref. [53]), which is however still higher than that of C_60_‐e‐BP (see Figure S13, Supporting Information). The increase of generated ·OH radicals under ultrasound stimulation is further confirmed by electron spin resonance spectroscopic characterizations (see Figure S14, Supporting Information). These results indicate that the covalent functionalization pattern of BPNSs sensitively affects their ·OH radical generation capability.

We further examined the possibility of other types of ROS generation. 9,10‐anthracene bis(methylene)‐dialdehyde acid (ABDA)^[^ ^54^ ^]^ and dihydroethidium (DHE)^[^ ^31^ ^]^ were used as the probe for detecting the singlet oxygen (^1^O_2_) and superoxide anion (O_2_^−^) radicals respectively. ABDA is a fluorescent molecule, which becomes non‐fluorescent when selectively oxidized by ^1^O_2_ radical.^[^ ^54^ ^]^ Interestingly, all samples show comparable fluorescence intensities to the control sample (pure ABDA solution), and negligible changes on the fluorescence intensity were observed after ultrasound excitation (Figure 3b and Figure S15, Supporting Information). This indicates that both pristine BPNSs and functionalized BPNSs cannot produce ^1^O_2_ radicals under our conditions, even after exposed to ultrasound. Likewise, upon changing the fluorescence probe to DHE for detecting the superoxide anion (O_2_^−^) radicals, all samples exhibit slight increases on the fluorescence intensity after ultrasound excitation (Figure 3c and Figure S16, Supporting Information), indicating that ultrasound excitation can induce only a small amount of O_2_^−^ radicals. Based on these results, we conclude that ·OH radicals are the dominant ROS produced by the pristine BPNSs and functionalized BPNSs after ultrasound excitation, and the highest amount of ·OH radicals can be achieved by BA‐s‐BP.

It has been reported that BP has cytotoxicity to both normal cells and tumor cells, and its cytotoxicity seems to be related to its size (see Table S3, Supporting Information).^[^ ^40^, ^41^ ^]^ We next examined the intrinsic cytotoxicity of the pristine and functionalized BPNSs in the absence of ultrasound exposure. Interestingly, after co‐cultured with mouse 4T1 breast tumor cells for 72 h, the pristine BPNSs show significant toxicity to detectable extent, whereas C_60_‐s‐BP, C_60_‐e‐BP, and BA‐s‐BP all exhibit negligible toxicity. Similar results are obtained for Hela cancer cells and NIH‐3T3 normal cells despite that the pristine BPNSs appear less toxic to these two types of cells (see Figure S17, Supporting Information). Therefore, covalent functionalization helps to inhibit the cytotoxicity of BPNSs.

**Figure 4:**
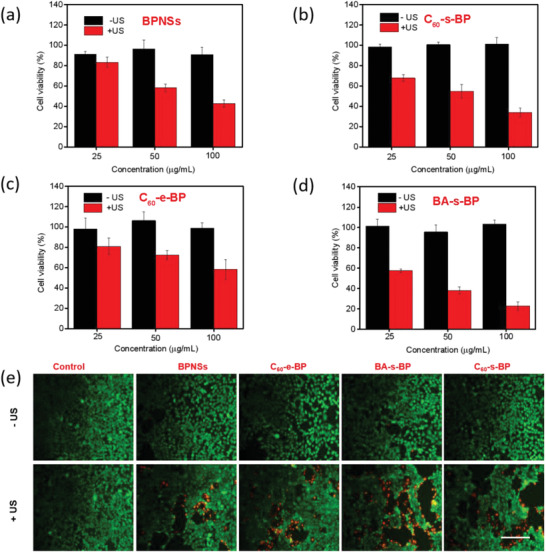
a–d) In vitro cytotoxicities of the pristine and functionalized BPNSs with different concentrations against 4T1 cells without (−US) and with ultrasound (+US) irradiation. e) Fluorescence microscopy images of 4T1 cells treated with the pristine and functionalized BPNSs (at 50 µg mL^−1^) in the presence of ultrasound exposure (at an output power density of 1.0 W cm^−2^ for 5 min). The control sample is FBS‐supplemented DMEM. Calcein‐AM (green) and PI (red) were used to label live and dead cells, respectively. Scale bar = 200 µm.

To systematically evaluate the SDT effect of the pristine and functionalized BPNSs, we carried out a series of in vitro and in vivo measurements. We first studied their abilities to kill mouse 4T1 breast tumor cells by using the Cell Counting Kit‐8 (CCK‐8) assay under ultrasonic conditions (**Figures** **4**a–d). In the absence of ultrasonic, the cell viability exceeds 90% even at a relatively high dose of 100 µg mL^−1^ for the pristine and functionalized BPNSs, indicating lack of inherent cytotoxicity within experimental time range (4 h). In sharp contrast, after ultrasonic exposure (1.0 W cm^−2^, 5 min), the pristine and functionalized BPNSs all show significant cytotoxicities, confirming that the pristine and functionalized BPNSs have SDT effects since ultrasound triggers their cytotoxicities. Moreover, the SDT efficacy of the functionalized BPNSs is sensitively dependent on the covalent functionalization pattern. Among C_60_‐s‐BP, C_60_‐e‐BP, and BA‐s‐BP, BA‐s‐BP shows the highest SDT efficacy under ultrasonic conditions, and the cell survival rate is reduced to ≈23% at a dose of 100 µg mL^−1^. Similar results were observed under a fluorescence microscope with dead/live staining by using cell‐permeable Calcein‐AM and cell‐impermeable propidium iodide (PI) to label live (green) and dead (red) cells respectively. As illustrated in Figure 4e, while a large number of living cells (green) appear for the pristine and functionalized BPNSs without ultrasonic, after ultrasonic excitation some cells are stained in red, indicating cell death. Among C_60_‐s‐BP, C_60_‐e‐BP, and BA‐s‐BP, the largest proportion of dead cells is observed for BA‐s‐BP, indicating its highest SDT efficacy. In contrast, ultrasonic itself results in almost no cell death according to the result of control sample of Fetal bovine serum (FBS)‐supplemented Dulbeccos modified Eagle's medium (DMEM). Therefore, these results confirm the dependence of the SDT efficacy of the functionalized BPNSs on the covalent functionalization pattern.

In order to confirm whether it is the intracellular ROS produced after ultrasonic excitation that causes cell death, we use cell‐permeable 2,7‐dichloro‐dihydrofluorescein diacetate (DCFH‐DA) as a probe to detect generation of the intracellular ROS. Clearly, after ultrasonic excitation the fluorescence intensity of DCFH‐DA at ≈525 nm increases dramatically for the pristine and functionalized BPNSs, suggesting that ultrasonic triggers generation of more intracellular ROS. Again, BA‐s‐BP exhibits the highest fluorescence intensity, suggesting that BA‐s‐BP generates the highest amount of intracellular ROS, which is increased by ≈2.2 times relative to that obtained without ultrasonic (Figure S18, Supporting Information). These results are consistent with those obtained from the comparative study of killing the 4T1 cells discussed above, confirming that the observed cytotoxicity of BPNSs is attributed to its sonodynamic‐stimulated ROS generation.

**Figure 5:**
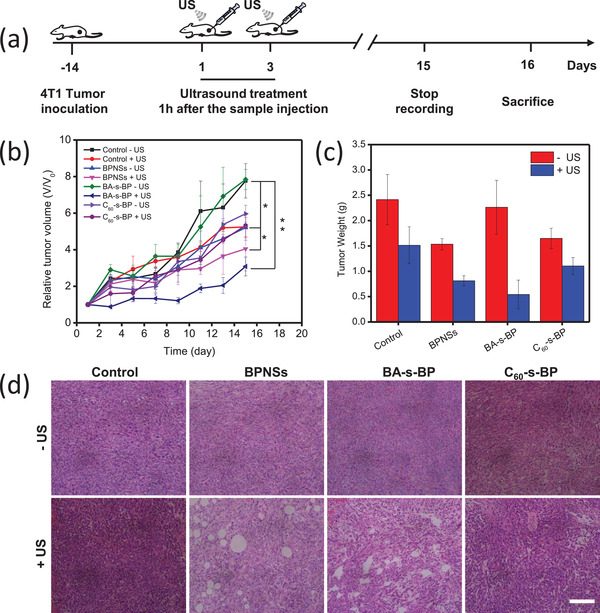
a) Schedule of the sonodynamic therapy. The pristine and functionalized BPNSs were injected intratumorally once a day (≈5 mg kg^−1^ per mouse) on days 1 and 3. Ultrasound exposure was applied 1 h after sample injection on days 1 and 3 with an ultrasound transducer (1 MHz) at an output power density of 2.5 W cm^−2^ for 10 min. Due to the inferior in vitro cytotoxicity revealed in the above study, C_60_‐e‐BP was not applied in this measurement. b) Changes of the average tumor volumes during the observation window of 15 days. Data points are reported as mean ± standard deviation (*n* = 5). c) The average tumor weights collected on day 15. Data points are reported as mean ± standard deviation (*n* = 5). d) Microscopic images of hematoxylin and eosin stained tissue slices of tumors collected on day 14. The tumors were treated by different samples with and without ultrasound exposure. Scale bar = 100 µm.

Encouraged by the in vitro sonodynamic cytotoxicity of BPNSs, we next assessed their antitumor SDT efficacy in vivo in mouse models. Briefly, 40 mice bearing 4T1 tumors were randomly divided into eight groups (*n* = 5 per group) to receive ultrasound or without ultrasound treatments (**Figure** **5**a). Clearly, the pristine and surface‐functionalized BPNSs can all inhibit tumor growth to a certain extent, while their efficiencies of inhibiting tumor growth are quite different (Figure 5b). Among them, BA‐s‐BP shows the best anti‐tumor effect under ultrasound conditions, with the tumor volume inhibition rate reaching 61% at the end of the therapy for 15 days. This is further confirmed by weighing the tumors collected from all groups, revealing that ultrasound excitation leads to inhibited tumor growth, while BA‐s‐BP exhibits the highest inhibition effect (Figure 5c). This phenomenon is consistent with the generation of the highest amount of intracellular ROS for BA‐s‐BP, which inhibits tumor growth effectively.

Finally, the systemic toxicity of BPNSs needs to be checked to ensure their biomedical safety for in vivo applications. As mentioned above, treatment of 4T1‐tumor‐bearing mice with the pristine and functionalized BPNSs can effectively inhibit tumor growth after ultrasound treatments, and no significant body‐weight changes are observed throughout the observation window (Figure S19, Supporting Information), indicating lack of acute toxicity. In addition, H&E staining analyses of the tumor slices from mice sacrificed at the end point of the therapeutic evaluation reveal that, after treatments by the pristine and functionalized BPNSs with ultrasound, the tumor slices exhibit apparent lesion and abnormality (Figure 5d). However, no noticeable abnormality is observed for the slices of major organs (heart, liver, spleen, lung, and kidney) (Figure S20, Supporting Information), confirming the negligible systemic toxicity of BPNSs.

To understand why BA‐s‐BP generates higher amount of ·OH radicals and consequently delivers higher antitumor SDT efficacy than C_60_‐s‐BP and C_60_‐e‐BP, we propose a plausible mechanism on the basis of their band energy level alignments. We measured the conduction band (CB) and valence band (VB) energy levels of the pristine BPNSs, C_60_‐s‐BP, C_60_‐e‐BP, and BA‐s‐BP by synchrotron radiation photoemission spectroscopy (SR‐PES). As reported in our previous paper, for the pristine BPNSs, ultrasonic excitation may induce its piezoelectric polarization, imposing positive shift of its VB edge, which becomes even more positive than the redox potential of H_2_O/OH (0.51 V versus RHE), consequently ·OH radicals are generated preferably as the major type of ROS killing tumors.^[^ ^31^ ^]^ After C_60_‐surface‐functionalization, the VB energy level of C_60_‐s‐BP (0.46 V versus RHE) shifts positively relative to that of the pristine BPNSs (0.40 V versus RHE), which becomes more close to the redox potential of H_2_O/OH (0.51 V versus RHE), thus the ultrasonic‐excitated piezoelectric polarization is more favorable, leading to generation of higher amount of ·OH radicals relative to the pristine BPNSs (see Figures S21–S23 and Table S4, Supporting Information). However, upon edge‐functionalization of BPNSs by C_60_, since the VB energy level of C_60_‐e‐BP (0.14 V versus RHE^[^ ^37^ ^]^) is much more negative than those of the C_60_‐s‐BP and the pristine BPNSs, hole transfer from the VB of C_60_‐e‐BP to a H_2_O molecule affording ·OH radicals becomes unfavorable energetically. As a result, C_60_‐e‐BP generates lower amount of ·OH radicals than C_60_‐s‐BP. On the other hand, although the VB energy level of BA‐s‐BP (0.22 V versus RHE) shifts negatively relative to that of the pristine BPNSs as well, its CB energy level also shifts negatively, which becomes more negative than the redox potential of O_2_/•O_2_^−^ (−0.33 V), energetically facilitating generation of •O_2_^−^, which further converts to ·OH radicals as the dominant ROS product to ablate tumors.^[^ ^55^ ^]^ In this sense, the inferior abilities of both C_60_‐s‐BP and C_60_‐e‐BP in generating ·OH radicals are primarily attributed to the radical scavenging property of C_60_.

## 3 Conclusion

In summary, covalently functionalized BPNSs are applied in SDT for the first time, resulting in boosted SDT efficacy along with inhibited cytotoxicity. On the basis of syntheses of a novel fullerene‐functionalized BPNSs with C_60_ covalently bonded onto the surface of BPNSs (C_60_‐s‐BP) as well as benzoic acid surface‐functionalized BPNSs (BA‐s‐BP) and C_60_ edge‐functionalized BPNSs (C_60_‐e‐BP), we systematically investigate the role of covalent functionalization pattern on the SDT efficacy and cytotoxicity of BPNSs. Except C_60_‐e‐BP, both surface‐functionalized BPNSs (C_60_‐s‐BP, BA‐s‐BP) exhibit higher SDT efficacies than the pristine BPNSs, while the highest SDT efficacy is achieved for BA‐s‐BP. Hydroxyl (·OH) radicals are identified to act as the dominant ROS to kill the tumor cells, and the highest SDT efficacy of BA‐s‐BP is interpreted by its strongest capability of generating ·OH radicals. Our study demonstrates for the first time that covalent functionalization is an effective approach to boost the SDT efficacy of BP in addition to improving its ambient stability, opening up a new avenue for biomedical applications of BP.

## 4 Experimental Section

### Materials

BP crystals were purchased from XFNANO (Nanjing, China). 4‐Azidobenzoin acid (97%), DCC, DMAP, sodium hydroxide (NaOH), and tetrabutylammonium hydroxide (TBAH, 40 wt% in water) were obtained from Sigma Aldrich. C_60_ (99.6%) was purchased from FUNANO (Xiamen, China). DMF and isopropanol (IPA) were obtained from Sinopharm Chemical Reagent Co. Ltd (Shanghai, China). All chemicals were used directly without further purification. DCFH‐DA and PTA were purchased from ShangHai YuanYe Biotechnology (Shanghai, China). CCK‐8 was obtained from Beyotime Institute of Biotechnology (Jiangsu, China). PBS (10×) saline was purchased from Beyotime Institute of Biotechnology (Jiangsu, China) and diluted by tenfold prior to use. Calcein AM, and propidium iodide (PI) were obtained from Life‐Technologies (ThermoFisher Scientific, USA). FBS was obtained from Shanghai ExCell Biology, Inc. (Shanghai, China). Trypsin (with EDTA, 0.25%) and modified DMEM were purchased from Hyclone. The 4T1 cells were purchased from the Cell Bank of the Chinese Academy of Sciences (Shanghai, China) and cultured in DMEM including 10% FBS at 37 °C in an incubator under a humidified atmosphere condition containing 5% CO_2_.

### Preparations of BPNSs and the Benzoic Acid Surface‐Functionalized BPNSs (BA‐s‐BP)

BPNSs were exfoliated in DMF from bulk BP. Briefly, 15 mg bulk BP was pound into powder in the glove box and then dispersed in 15 mL DMF. The BP dispersion sealed well in the sample vial was then sonicated at low temperature for 20 h. Afterward, the resultant brown suspension was prepared and then centrifuged at 2000 rpm for 15 min to remove the residual un‐exfoliated big particles, yielding brown nanosheets supernatant (BPNSs dispersed in DMF).

BA‐s‐BP was synthesized according to the procedure reported previously.^[^ ^34^ ^]^ Briefly, BPNSs (5 mg, 0.161 mmol) dispersed in DMF and 4‐azidobenzoic acid (32 mg, 0.192 mmol) were mixed in a round‐bottom two‐necked flask equipped with tee and a balloon. Then the mixture was deaerated repeatedly using evacuation and filling with argon at least six times, and heated up to the 140 °C for 48 h with strong stirring. The suspension was centrifuged at 12 000 rpm for 30 min to obtain the enriched nanosheets over the reaction. Then the sample was washed by DMF for three times to remove the unreacted 4‐azidobenzoin acid. The obtained residue was used directly for the next reaction without drying in a vacuum oven.

### Synthesis of Fullerenol (C_60_(OH)*_n_*)

A toluene solution of C_60_ (215 mg in 100 mL) was vigorously stirred with aqueous NaOH (6 g in 6 mL water). Then 8 drops of TBAH (40% in water) were added to the solution drop by drop. The solution was stirred at room temperature under air for 3 h and then the toluene solution, originally deep violet, turned colorless and a brown sludge precipitation. Then 100 mL methanol was added and then the suspension was centrifuged at 10 000 rpm for 10 min to obtain the residue. Then the synthetic fullerenol was washed by methanol and water for several times to remove raw materials and TBAH, forming fullerenol soluble in DMF. The obtained fullerenol was then dried in a vacuum oven at 50 °C for 24 h.

### Preparation of C_60_‐s‐BP

BA‐s‐BP (5 mg) dispersed in DMF and fullerenol C_60_(OH)*_n_* (10 mg) were mixed in a round‐bottom two‐necked flask equipped with tee and a balloon. DCC (111 mg) and DMAP (24 mg) were added to the solution. The mixture was deaerated repeatedly via evacuation and filling with argon, and then heated to 110 °C for 3 days with intense stirring. When the reaction was over, the suspension was centrifuged at 12 000 rpm for 32 min to obtain the residue. Then the residue was washed by DMF for three times to remove unreacted fullerenol, DCC, and DMAP, and further washed by IPA for three times to remove DMF. The obtained sample was then dried in a vacuum oven at 50 °C for 48 h.

### ROS Generation by Ultrasound Irradiation

The DCFH‐DA (1 mm, 100 µL in DMSO) was mixed with NaOH (0.01 m, 400 µL in Millipore water) in dark, and the resulting mixture was left standing still for 30 min and then addition of NaH_2_PO_4_ (25 mm, 2 mL in Millipore water), which yielded DCFH solution (40 µm assuming 100% conversion of DCFH‐DA to DCFH). The DCFH solution (40 µm, 100 µL) was added into BPNSs or different ‐functionalized BPNSs (BA‐s‐BP, C_60_‐e‐BP, and C_60_‐s‐BP) dispersions (100 µg mL^−1^, 100 µL in PBS), treated by ultrasound for 10 min (1.0 MHz, 1.0 W cm^−2^) by ultrasonic transducer (WELLD, WED‐100). There was no stop interval during the ultrasound process, and the ultrasound time of each group remained same. Different samples mixed with DCFH without ultrasound exposure were as a control. To exclude the background fluorescence of DCFH induced by ultrasound, control PBS sample with and without ultrasound exposure were also used. The samples dispersion was centrifuged at 10 000 g for 10 min (Eppendorf, Centrifuge 5810 R) after treating with or without ultrasound, and the fluorescence spectra of resulting supernatants were monitored with a fluorometer (F‐4600 spectrofluorometer, Hitachi) (*λ*_ex_ = 480 nm, *λ*_em_ = 500–700 nm, slit widths were 5.0 nm for both excitation and emission wavelengths).

PTA solution (1000 µm, 100 µL in 2 mm NaOH aqueous solution) was added into different BPNSs dispersions (100 µg mL^−1^, 100 µL in PBS), treated with ultrasound for 10 min (1.0 MHz, 1.0 W cm^−2^) using ultrasonic transducer (WELLD, WED‐100). To exclude the background fluorescence of PTA, the fluorescence of PBS mixed with PTA under ultrasound condition or not for 10 min was monitored. After treatment for 10 min, the resulting dispersions were centrifuged (Eppendorf, Centrifuge 5810 R) at 10 000 g for 10 min, and then the fluorescence spectra were monitored with a fluorometer (F‐4600 spectrofluorometer, Hitachi) (*λ*_ex_ = 315 nm, *λ*_em_ = 320–500 nm, slit widths were 5.0 nm and 20.0 nm for excitation and emission wavelengths, respectively).

ABDA solution (100 µL, 40 µm in PBS) was added into different BPNSs dispersions (100 µg mL^−1^, 100 µL in PBS), exposed with ultrasound for 10 min (1.0 MHz, 1.0 W cm^−2^) using ultrasonic transducer (WELLD, WED‐100). To ensure the influence of ultrasound treatment on the ROS generation by different samples (including the pristine BPNSs, BA‐s‐BP, C_60_‐e‐BP, and C_60_‐s‐BP), similar mixture of ABDA and different samples were prepared as described above. To exclude the background fluorescence of ABDA, the fluorescence of PBS mixed with ABDA under ultrasound condition or not for 10 min was monitored. After treatment for 10 min, the resulting dispersions were centrifuged (Eppendorf, Centrifuge 5810 R) at 10 000 g for 10 min, and then the fluorescence spectra were monitored with a fluorometer (F‐4600 spectrofluorometer, Hitachi) (*λ*_ex_ = 380 nm, *λ*_em_ = 400–600 nm, slit widths were 2.5 and 5.0 nm for excitation and emission wavelengths, respectively).

DHE could be oxidized by singlet oxygen to show green fluorescence. DHE solution (100 µL, 100 µm in PBS) was added into different BPNSs dispersions (100 µg mL^−1^, 100 µL in PBS), exposed with ultrasound for 10 min (1.0 MHz, 1.0 W cm^−2^) using ultrasonic transducer (WELLD, WED‐100). To ensure the influence of ultrasound treatment on the ROS formation by different samples (including the pristine BPNSs, BA‐s‐BP, C_60_‐e‐BP, and C_60_‐s‐BP), similar mixture of ABDA and different samples without ultrasound treatment were prepared as described above. To exclude the background fluorescence of ABDA, the fluorescence of PBS mixed with ABDA under ultrasound condition or not for 10 min was monitored. After treatment for 10 min, the resulting dispersions were centrifuged (Eppendorf, Centrifuge 5810 R) at 10 000 g for 10 min, and then the fluorescence spectra were monitored with a fluorometer (F‐4600 spectrofluorometer, Hitachi) (*λ*_ex_ = 480 nm, *λ*_em_ = 500–700 nm, slit widths were 10.0 and 20.0 nm for excitation and emission wavelengths, respectively).

### CCK‐8 Viability Assays

CCK‐8 assays were carried out to determine the cytotoxicity of BPNSs, BA‐s‐BP, C_60_‐e‐BP, and C_60_‐s‐BP using murine 4T1 cells, Hela cells, and NIH‐3T3 cells. Each well of a 96‐well microplate was seeded with ≈10^4^ cells, incubated at 37 °C for 24 h till up to ≈80% confluency. Then adding different sample dispersions (100 µL in FBS‐supplemented DMEM) into the resulting cell culture to an expected concentration (50 µg mL^−1^), incubated at 37 °C for 24 h, the dispersions with different BP samples were removed and washed twice with PBS, replenished with fresh FBS‐supplemented DMEM (200 µL) and then continuous incubated for 24 or 48 h. The resulting culture was subsequently washed with sterile PBS, replenished with fresh FBS‐supplemented DMEM (100 µL), incubated in dark for another 2 h (37 °C, 5% CO_2_), and then assayed for cell viability with a CCK‐8 kit by adding 10 µL CCK‐8 into a well of a 96‐well microplate and then incubating for 45 min (37 °C, 5% CO_2_). Cell viability was quantified by measuring the optical density at 450 nm (OD450) using a microplate reader (Varioskan, Thermo). Cell viability ratio was referred as the relative ratio of the OD450 of treated cells to that of the control. Each trial was performed in triplicate and the reported results were averages of two independent trials.

CCK‐8 assays were carried out to determine how ultrasound treatment affects the cytotoxicity of the pristine BPNSs, BA‐s‐BP, C_60_‐e‐BP, and C_60_‐s‐BP using murine 4T1 breast cancer cell‐lines. Each well of a 96‐well microplate was seeded with ≈10^4^ cells, incubated at 37 °C for 24 h till up to ≈80% confluency. Then adding different sample dispersions (100 µL in FBS‐supplemented DMEM) into the resulting cell culture to an expected concentration (25, 50, 100 µg mL^−1^), incubated at 37 °C for 4 h, the dispersions with different BPNSs samples were removed and washed twice with PBS, replenished with fresh FBS‐supplemented DMEM (200 µL) and then 5 min treatment with ultrasound (1.0 MHz, 1.0 W cm^−2^) using a ultrasonic transducer (WELLD, WED‐100). The resulting culture was subsequently washed with sterile PBS, replenished with fresh FBS‐supplemented DMEM (100 µL), incubated in dark for another 2 h (37 °C, 5% CO_2_), and then assayed for cell viability with a CCK‐8 kit by adding 10 µL CCK‐8 into a well of a 96‐well microplate and then incubating for 45 min (37 °C, 5% CO_2_). Cell viability was quantified by measuring the optical density at 450 nm (OD450) using a microplate reader (Varioskan, Thermo). Cell viability ratio was referred as the relative ratio of the OD450 of treated cells to that of the control. Each trial was performed in triplicate and the reported results were averages of two independent trials.

### Live/Dead Cell Viability Assays

Live/Dead cell viability assays under fluorescence microscopy (IX71, Olympus) were carried out to further detect how ultrasound treatment affects the cytotoxicity of different samples, using murine 4T1 breast cancer cell‐lines. 35‐mm Cell culture dish was seeded with ≈2 × 10^5^ cells in FBS‐supplemented DMEM (v/v 10%), then incubated for 24 h at 37 °C (5% CO_2_), removed supernatant, addition of different sample dispersions (50 µg mL^−1^, 1.0 mL in FBS‐supplemented DMEM), and then incubated for 4 h (37 °C, 5% CO_2_). The resulting culture was subsequently washed with sterile PBS, replenished with fresh FBS‐supplemented DMEM, the resulting cell culture was subsequently irradiated by ultrasound (1.0 MHz, 1.0 W cm^−2^) for 3 min. The resulting culture was subsequently washed with sterile PBS. Adding mixture of Calcein‐AM solution and PI (500 µL in PBS, to reach final concentrations of 2 and 4.5 µm for Calcein‐AM solution and PI, respectively) to the resulting culture, following incubated at 37 °C (5% CO_2_) for 15 min in dark, washed with sterile PBS twice, and then imaged by an inverted fluorescence microscope ((IX71, Olympus).

### Intracellular ROS Detection

≈2 × 10^5^ 4T1 cells were seeded into a 35‐mm cell culture dish, followed by incubation for 24 h at 37 °C (5% CO_2_). Supernatant of the resulting culture was subsequently removed, followed by addition of BPNSs dispersion (50 µg mL^−1^, 1.0 mL in 10% FBS‐supplemented DMEM) and 4‐h incubation (37 °C, 5% CO_2_). The mass of BPNSs injected in mouse was the same for the four groups of mice. When the 4‐h incubation was done, the supernatant was removed and the cells were gently washed twice with sterile PBS. The as‐washed cells were then redispersed into DCFH‐DA solution (10 µm, 1.0 mL in 10% FBS‐supplemented DMEM) and cultured (37 °C, 5% CO_2_) for 1 h, followed by supernatant removal, gentle wash twice with sterile PBS, and redispersing into 10% FBS‐supplemented DMEM. The resulting cell dispersion was then treated with ultrasound radiation (1.0 MHz, 1.0 W cm^−2^) for 5 min, washed twice with sterile PBS, followed by lysing with cell lysis buffer (500 µL) in dark for 12 h, and then the fluorescence spectra of resulting supernatants were monitored with a fluorometer (F‐4600 spectrofluorometer, Hitachi) (*λ*_ex_ = 480 nm, *λ*_em_ = 500–700 nm, slit widths were 5.0 nm for both excitation and emission wavelengths).

### In Vivo SDT Cancer Treatment

To construct the tumor model, mice received subcutaneous injection of 3 × 10^6^ 4T1 cells on the back. And inoculated for 14 days. After the tumor volume reached 150 mm^3^, the mice were randomly divided into eight groups (*n* = 5 in each group): 1) PBS as control, 2) the pristine BPNSs, 3) BA‐s‐BP, 4) C_60_‐s‐BP, 5) +US, 6) BPNSs (+US), 7) BA‐s‐BP (+US), 8) C_60_‐s‐BP (+US). 50 µL of saline containing BP (2 mg mL^−1^) or BA‐s‐BP and C_60_‐s‐BP (with BP concentration at 2 mg mL^−1^) were intratumorally injected (≈5mg kg^−1^ per mouse) on days 1 and 3. The mice were irradiated under ultrasound (1 MHz, 2.5 W cm^−2^) for 10 min (1 h after the sample injection on days 1 and 3), and 3 mm pig skin was added between ultrasound probe and tumor. The tumor volume as well as body weight was recorded every other day. The sample was injected intratumorally once a day. On day 16, all the mice were euthanized, and the major organs (heart, liver, spleen, lung, and kidney) as well as tumors were excised for histopathologic study. The tumor size was measured by a Vernier caliper regularly and tumor volume (*V*) was calculated by the following formula: *V* = (tumor length) × (tumor width)^2^/2. All animal experiments were conducted in compliance with the guidelines for the care and use of research animals established by the Animal Care and Use Committee at the University of Science and Technology of China.

### Characterizations

FTIR spectra were carried out on a TENSOR 27 spectrometer (Bruker, Germany) at room temperature. UV–vis absorption spectroscopy was performed on a 3700 UV–vis spectrometer (Shimadzu, Japan). Raman spectra were carried out in Via Raman Microscope equipment (Renishaw, England) with a 532 nm excitation laser. Scanning electron microscopy (SEM) images were performed using a JEOL JSM‐6390LA instrument (Rigaku, Japan). The HR‐TEM was obtained from a JEOL‐2010 (Rigaku, Japan) microscope operating at a voltage of 200 kV. X‐ray photoelectron spectroscopy (XPS) was performed on a Thermo‐VV ESCALAB 250 (Thermo‐VV Scientific) machine. AFM measurements were performed on a XE7 scanning probe microscope (Park, Korea). X‐ray diffraction was obtained from a Smart Lab 9 kW X‐ray diffraction instrument (Rigaku, Japan). Solid‐state 31P nuclear magnetic resonance spectra were carried out on a AVANCE AV 400 magic angle spinning (MAS) measurements (Bruker, Germany) using a standard Bruker 4 mm MAS probe with spinning speed 14 kHz. Elemental analysis was carried out on Elemental Analyzer (mode CHN), Germany Elementar Vario EL cube (Combustion tube: 950 °C, reduction tube: 550 °C). SR‐PES experiments were performed at the Catalysis and Surface Science endstation in the National Synchrotron Radiation Laboratory, Hefei China, measured using synchrotron radiation light as the excitation source with a photon energy of 39.9 eV. A sample bias of −5 V was applied to observe the secondary electron cutoff. The powder samples were fixed on the substrate with conductive adhesive and then loaded into the cavity and evacuated overnight before measurement.

## Conflict of Interest

The authors declare no conflict of interest.

## Supporting information

Supporting Information

## Data Availability

Research data are not shared.
