# Supplementary material for: Boosting Antitumor Sonodynamic Therapy Efficacy of Black Phosphorus via Covalent Functionalization
Source: Adv Sci (Weinh). 2021 Aug 13;8(20):2102422. doi: 10.1002/advs.202102422 (PMC8529424; doi:10.1002/advs.202102422)
Supplement: Supplementary file 1 — Supporting Information [file ADVS-8-2102422-s001.pdf]

© 2021 Wiley-VCH GmbH

ADVANCED SCIENCE
Open Access

Supporting Information

for *Adv. Sci.*, DOI: 10.1002/advs.202102422

Boosting Antitumor Sonodynamic Therapy Efficacy of Black Phosphorus via Covalent Functionalization

Yajuan Liu, Zhiyuan Li, Feng Fan, Xianjun Zhu, Lingbo Jia, Muqing Chen, Pingwu Du, Lihua Yang, and Shangfeng Yang**

Copyright WILEY-VCH Verlag GmbH & Co. KGaA, 69469 Weinheim, Germany, 2013.

Supporting Information

Boosting Antitumor Sonodynamic Therapy Efficacy of Black Phosphorus via Covalent Functionalization

Yajuan Liu, Zhiyuan Li, Feng Fan, Xianjun Zhu, Lingbo Jia, Muqing Chen, Pingwu Du, Lihua Yang, and Shangfeng Yang**

Contents

S1. Reaction scheme and chemical structure of BA-s-BP. 2
S2. Reaction scheme and chemical structure of $C_{60}(OH)_n$. 2
S3. The proportion of peak areas from C1s XPS spectrum of $C_{60}(OH)_n$. 3
S4. FT-IR spectra of C_{60}, $C_{60}(OH)_n$, BPNSs and BA-s-BP. 3
S5. XPS survey spectra of C_{60}-s-BP and BA-s-BP. 4
S6. High-resolution P2p XPS spectra of BA-s-BP and C_{60}-s-BP. 4
S7. Raman spectra of C_{60}-s-BP, BA-s-BP/$C_{60}(OH)_n$ mixture, $C_{60}(OH)_n$, C_{60}, BA-s-BP and BPNSs. 5
S8. Elemental analysis measurement of the C_{60}-s-BP. 6
S9. SEM images of the pristine BPNSs, BA-s-BP , $C_{60}(OH)_n$, and C_{60}-s-BP. 7
S10. TEM images of BA-s-BP and $C_{60}(OH)_n$. 7
S11. AFM images and the thickness distributions of BA-s-BP and C_{60}-s-BP. 8
S12. HR-TEM image of BA-s-BP 8
S13. XRD patterns of C_{60}-s-BP, $C_{60}(OH)_n$ and BA-s-BP. 9
S14. Stabilities of the pristine BPNSs and C_{60}-s-BP dispersions in water. 10
S15. Mechanism of PTA detecting ·OH radicals and Fluorescence emission spectra obtained by using PTA as the ·OH probe. 11
S16. ESR spectra of DMPO/·OH obtained from different samples. 12
S17. Reaction of ABDA with $^1O_2$ radicals and fluorescence emission spectra obtained by using ABDA as the $^1O_2$ probe. 13
S18. Reaction of DHE with $·O_2^-$ radicals and fluorescence emission spectra obtained by using DHE as the $·O_2^-$ probe. 14
S19. In vitro cytoxicity of BP to different cells reported in the literature 15
S20. In vitro cytotoxicity of different samples against 4T1, Hela and NIH-3T3 cells at different incubation times. 15
S21. Detecting the intracellular ROS with DCFH-DA. 16
S22. The average mouse body weights through the observation window. 17
S23. The H&E stained tissue slices of five main organs (Heart, liver, spleen, lung, Kidney) collected on day 16 of treatment. 17
S24. Energy level diagrams of the pristine and functionalized BPNSs. 18
References 21

S1. Reaction scheme and chemical structure of BA-s-BP.

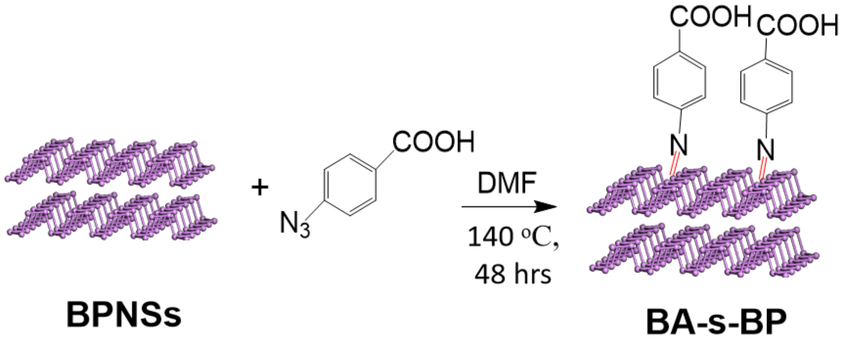

Figure S1. Reaction scheme and chemical structure of BA-s-BP. Copied from ref. 1.

S2. Reaction scheme and chemical structure of $C_{60}(OH)_n$.

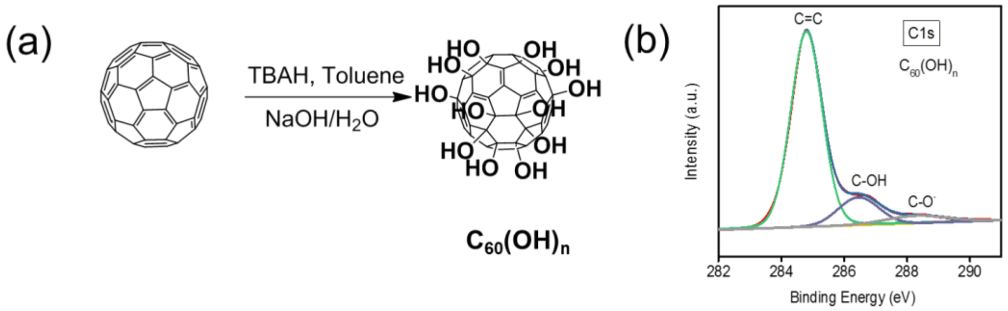

Figure S2. (a) Reaction scheme and chemical structure of $C_{60}(OH)_n$. (b) High-resolution C1s XPS spectrum of $C_{60}(OH)_n$.

S3. The proportion of peak areas from C1s XPS spectrum of $C_{60}(OH)_n$.

Table S1: The proportion of peak areas from C1s XPS spectrum of $C_{60}(OH)_n$.

Peak	1	2	3
Position	284.8 eV	286.5	288.3
Peak area	100778.1	17686.3	7043.1
proportion	80.3%	14.1%	5.6%
Assignment	C-C/C=C	C-OH	$C\text{-}O^-$

According to the integrated peak areas of different groups in C1s XPS spectrum, the proportions of C atoms within C-OH bonds and $C\text{-}O^-$ bonds are ~14.1% and ~5.6 % respectively, accordingly the number of hydroxyl (-OH) groups in the fullerenols (n) was calculated to be:

n=60 × (14.1% + 5.6%)=12, Where 60 refers to the number C atoms of C_{60}.

S4. FT-IR spectra of C_{60}, $C_{60}(OH)_n$, BPNSs and BA-s-BP.

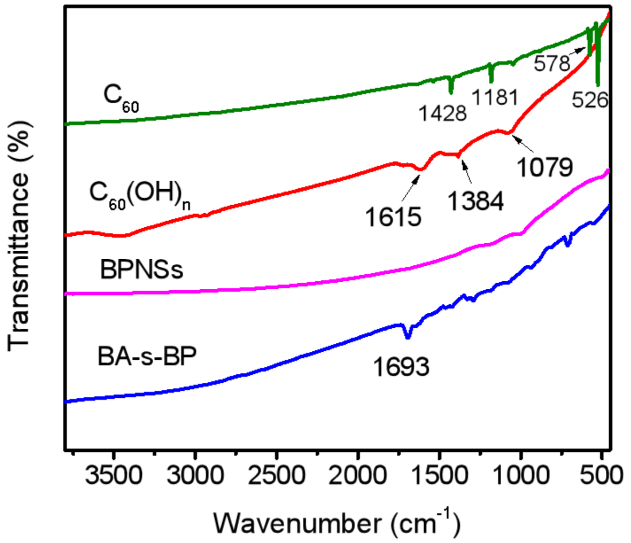

Figure S3. (a) FT-IR spectra of C_{60}, $C_{60}(OH)_n$, BPNSs and BA-s-BP. The FTIR spectrum of the C_{60} shows four characteristic vibrational peaks at 526, 578, 1181, and 1428 cm^{-1}. However, the four characteristic vibrational peaks disappeared after hydroxylation of C_{60} affording $C_{60}(OH)_n$ fullerenol because of the reduced cage symmetry, showing three characteristic peaks at 1615, 1384, and 1079 cm^{-1}. Compared to the pristine BPNSs, a new peak ascribed to –COOH at 1693 cm^{-1} appeared in the FT-IR spectra of BA-s-BP, verifying the successful covalent azide functionalization of BPNSs.

S5. XPS survey spectra of C_{60}-s-BP and BA-s-BP.

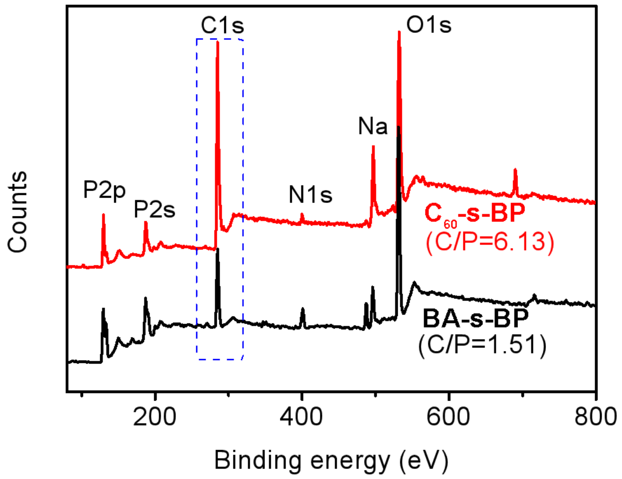

Figure S4. XPS survey spectra of C_{60}-s-BP and BA-s-BP.

S6. High-resolution P2p XPS spectra of BA-s-BP and C_{60}-s-BP.

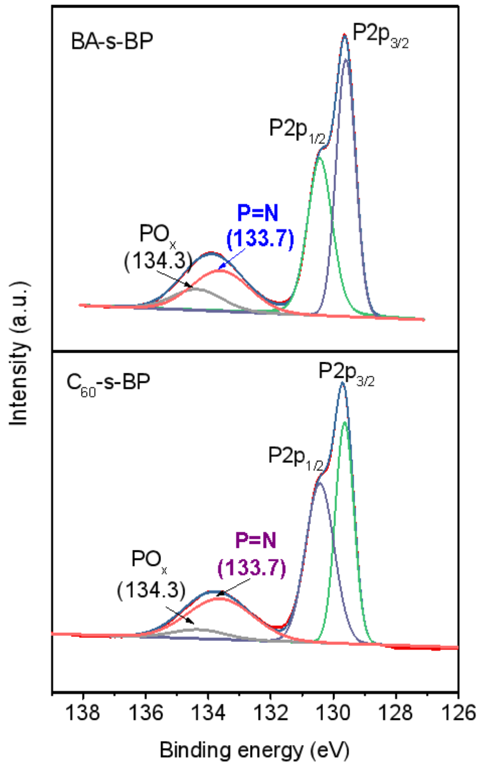

Figure S5. High-resolution P2p XPS spectra of BA-s-BP and C_{60}-s-BP. The spectrum of BA-s-BP was copied from ref. 1.

S7. Raman spectra of C_{60}-s-BP, BA-s-BP/$C_{60}(OH)_n$ mixture, $C_{60}(OH)_n$, C_{60}, BA-s-BP and BPNSs.

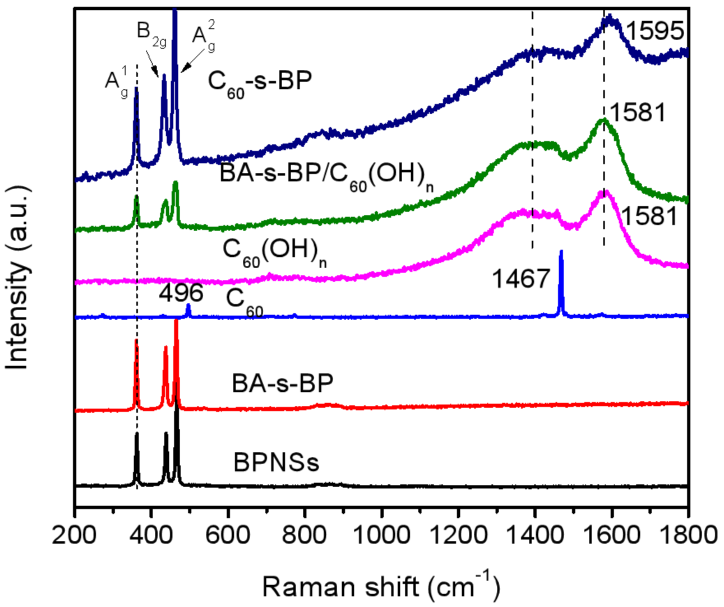

Figure S6. Raman spectra of C_{60}-s-BP, BA-s-BP/$C_{60}(OH)_n$ mixture, $C_{60}(OH)_n$, C_{60}, BA-s-BP and BPNSs.

There are two intense peaks at 496 and 1467 cm^{-1} in the Raman spectrum of C_{60}. However, the peak at 496 cm^{-1} disappeared and the peak at 1467 cm^{-1} was broadened in $C_{60}(OH)_n$ because of reduced symmetry. Instead, a new peak at 1581 cm^{-1} ascribed to fullerene cage appeared, which shifted positively by ~14 cm^{-1} after covalent functionalization with BP. On the other hand, the Raman characteristic peaks of BA-s-BP/$C_{60}(OH)_n$ mixture were simply a superposition of BA-s-BP and $C_{60}(OH)_n$. These results prove the successful covalent bonding of C_{60} onto BPNSs. The Raman spectra of BPNSs and BA-s-BP were copied from ref. 1.

S8. Elemental analysis measurement of the C_{60}-s-BP.

Table S2. Elemental analysis measurement of the C_{60}-s-BP.

Sample	Weight (mg)	N (%)	C (%)	H (%)
1	1.3070	0.05	24.52	0.045
2	1.0300	0.03	23.52	0.07
3	1.3710	1.47	21.84	0.055
average		0.51	23.29	0.057

According to the weight percentage of C element of 23.29 %, the molar ratio of C_{60} and P atoms can be calculated by:

$$m\% = \frac{23.29\% * \frac{1}{12} * 1/67}{((1 - 23.29\% * \frac{1}{12} * \frac{1}{67} * 720)/31)} = 1.13\%$$

Where 12 and 31 are the atomic weights of C and P atoms, respectively, 67 is the number of carbons of a fullerene group grafting to the surface of BP, and 720 is the molecular weight of C_{60}.

S9. SEM images of the pristine BPNSs, BA-s-BP , $C_{60}(OH)_n$, and C_{60}-s-BP.

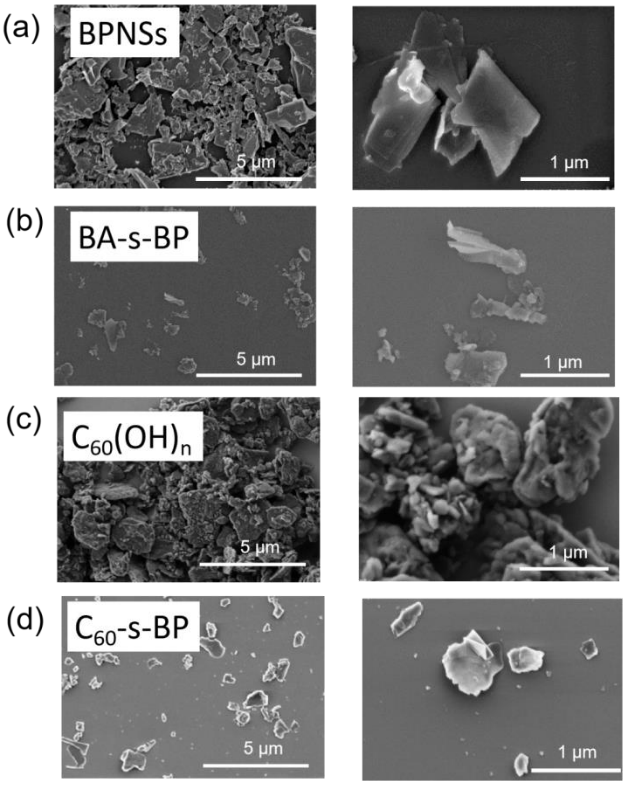

Figure S7. SEM images of the pristine BPNSs (a), BA-s-BP (b), $C_{60}(OH)_n$ (c), and C_{60}-s-BP (d) at different magnifications. Images of BPNSs and BA-s-BP were copied from ref. 1

S10. TEM images of BA-s-BP and $C_{60}(OH)_n$.

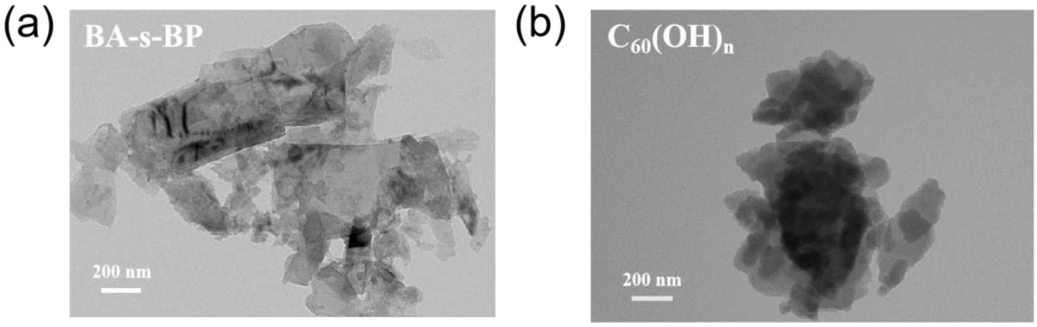

Figure S8. TEM images of BA-s-BP and $C_{60}(OH)_n$. Image of BA-s-BP was copied from ref. 1.

S11. AFM images and the thickness distributions of BA-s-BP and C_{60}-s-BP.

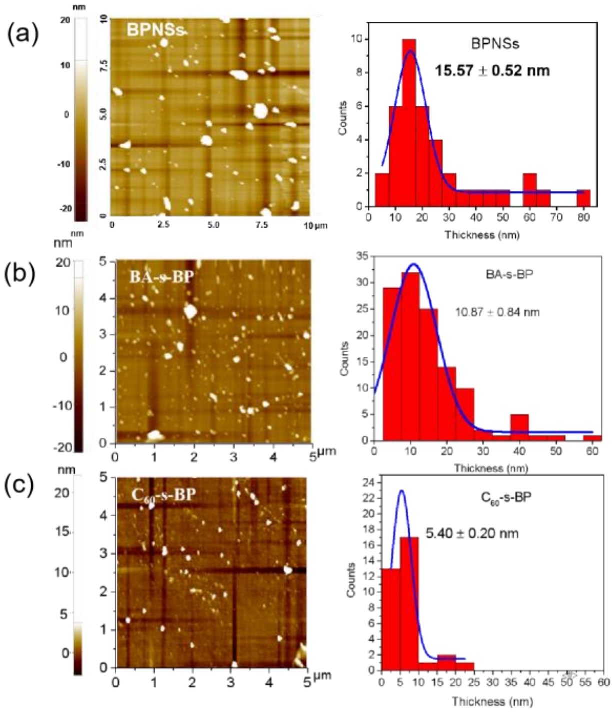

Figure S9. AFM images (a, c) and the thickness distributions (b, d) of the pristine BPNSs (a), BA-s-BP (b) and C_{60}-s-BP (c). The data of the pristine BPNSs and BP-s-BP were copied from ref. 1.

S12. HR-TEM image of BA-s-BP

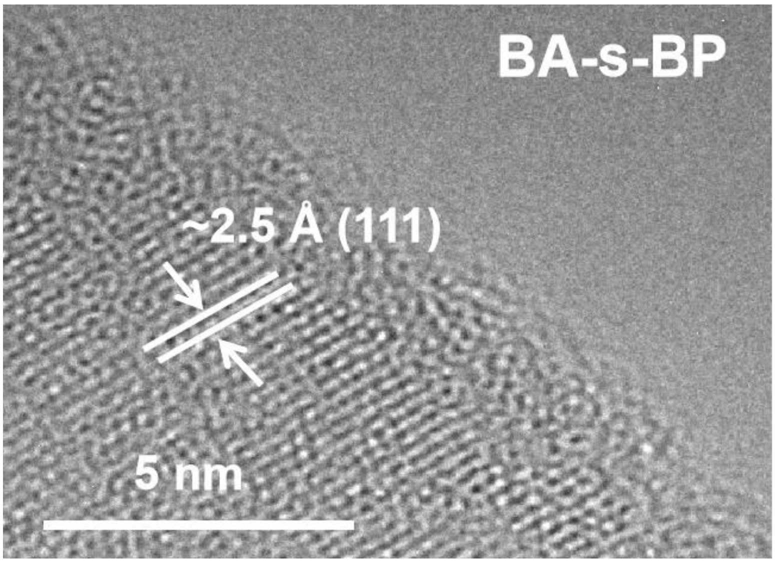

Figure S10. HR-TEM image of BA-s-BP (copied from ref. 1).

S13. XRD patterns of C_{60}-s-BP, $C_{60}(OH)_n$ and BA-s-BP.

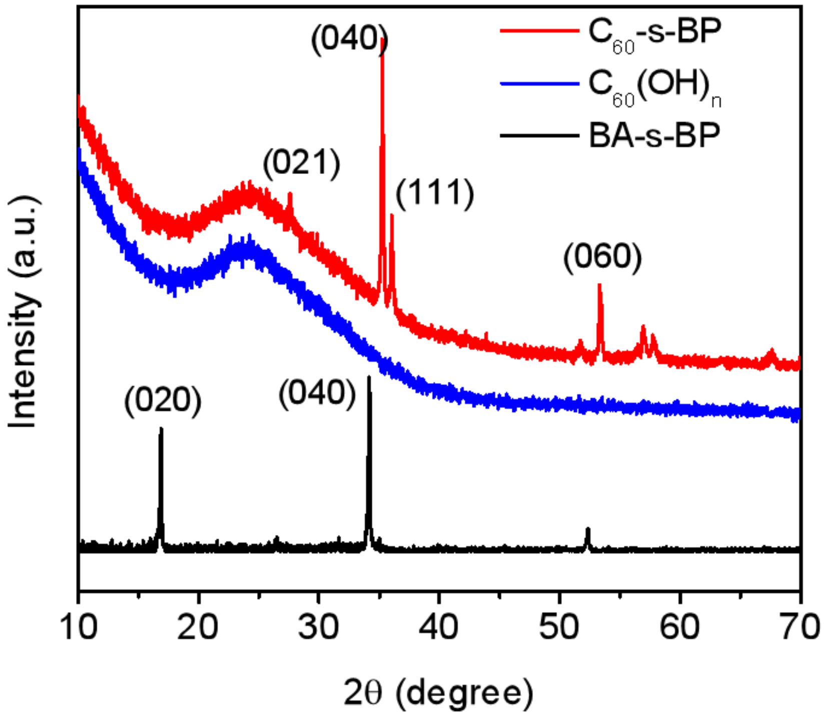

Figure S11. XRD patterns of C_{60}-s-BP, $C_{60}(OH)_n$ and BA-s-BP. XRD data of BA-s-BP was copied from ref. 1.

S14. Stabilities of the pristine BPNSs and C_{60}-s-BP dispersions in water.

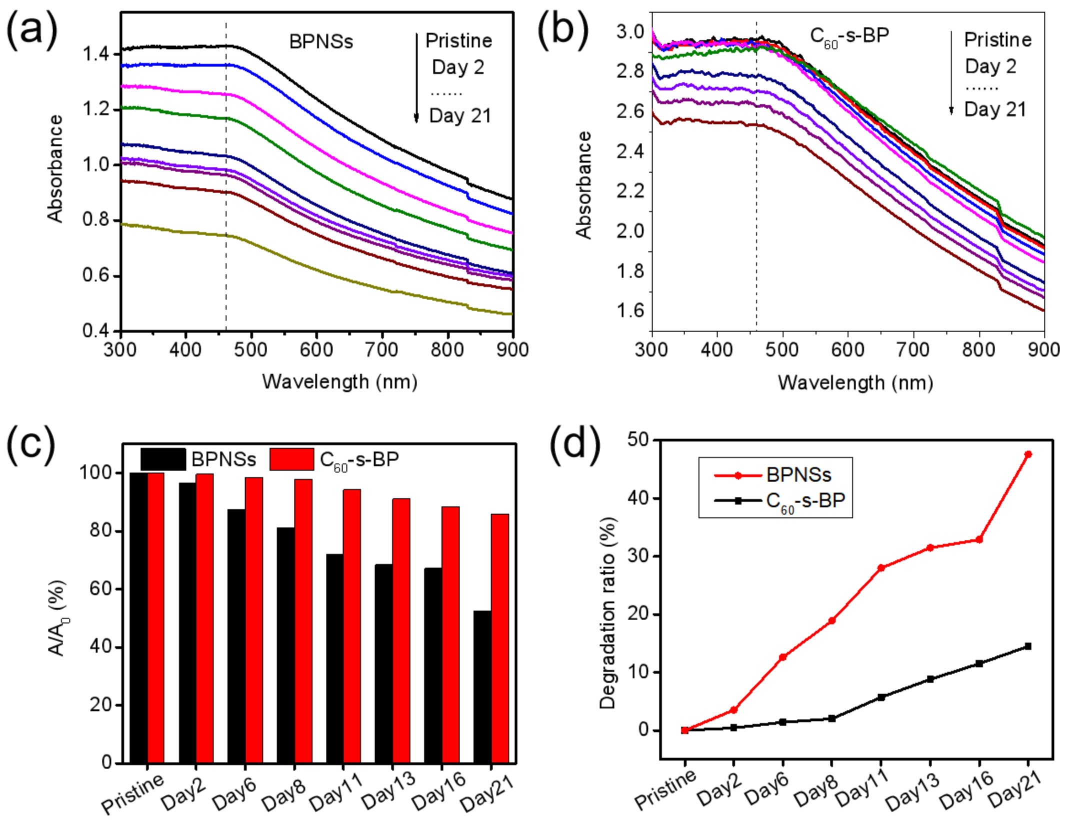

Figure S12. Stabilities of the pristine BPNSs and C_{60}-s-BP dispersions in water. UV-vis absorption spectra of the pristine BPNSs (a) and C_{60}-s-BP (b) dispersed in water after standing for different times. (c) Change of the absorption ratios (A/A_0) at 460 nm of the pristine BPNSs and C_{60}-s-BP dispersions with different times. (d) Degradation ratios ($1-A/A_0$) of the pristine BPNSs and C_{60}-s-BP after exposure to air for 21 days. The stability data of the pristine BPNSs was copied from ref. 1.

S15. Mechanism of PTA detecting •OH radicals and Fluorescence emission spectra obtained by using PTA as the •OH probe.

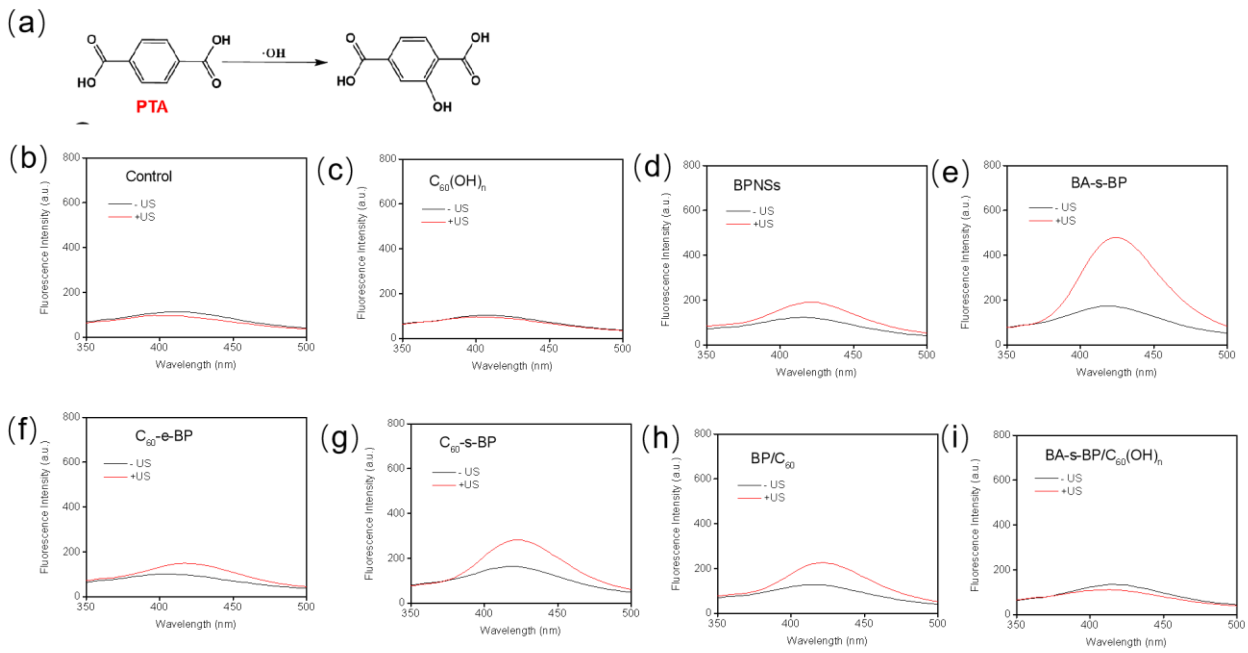

Figure S13. (a) Mechanism of PTA detecting •OH radicals. (b-i) Fluorescence emission spectra obtained by using PTA as the •OH probe. -US refers to the presence of 50 μg/mL of BP nanosheet in PBS solution without ultrasound irradiation, and +US refers to ultrasound excitation at the output power density of 1.0 W/cm^2, 1.0 MHz.

S16. ESR spectra of DMPO/·OH obtained from different samples.

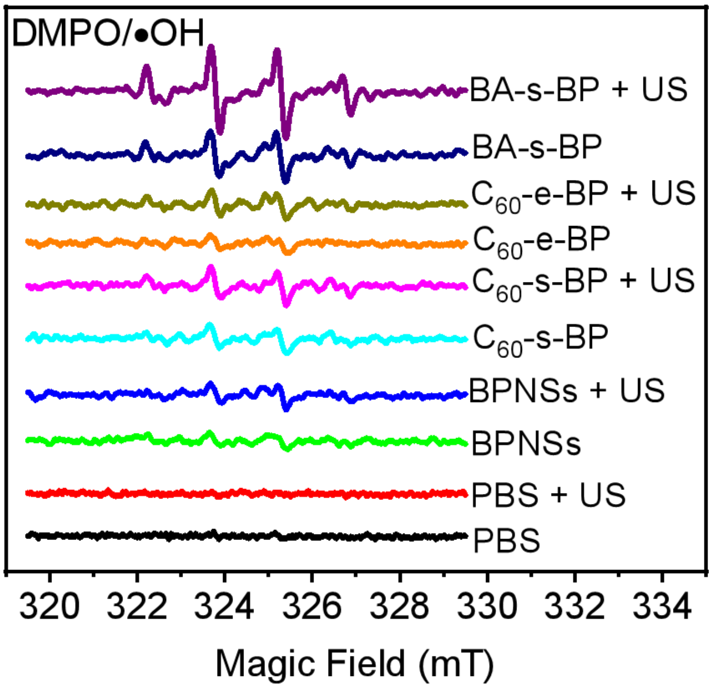

Figure S14. ESR spectra of DMPO/·OH of different samples (100 μg/ml BP nanosheets in PBS) in the presence of ultrasound exposure (at 1.5 W/cm^2 for 10 min) (" sample name+ US"), with those in the absence of ultrasound exposure included as references. PBS in the presence ("PBS + US") and absence ("PBS") of ultrasound exposure (at 1.5 W/cm^2 for 10 min) were included for comparison. DMPO as a scavenger ·OH radicals.

S17. Reaction of ABDA with $^1O_2$ radicals and fluorescence emission spectra obtained by using ABDA as the $^1O_2$ probe.

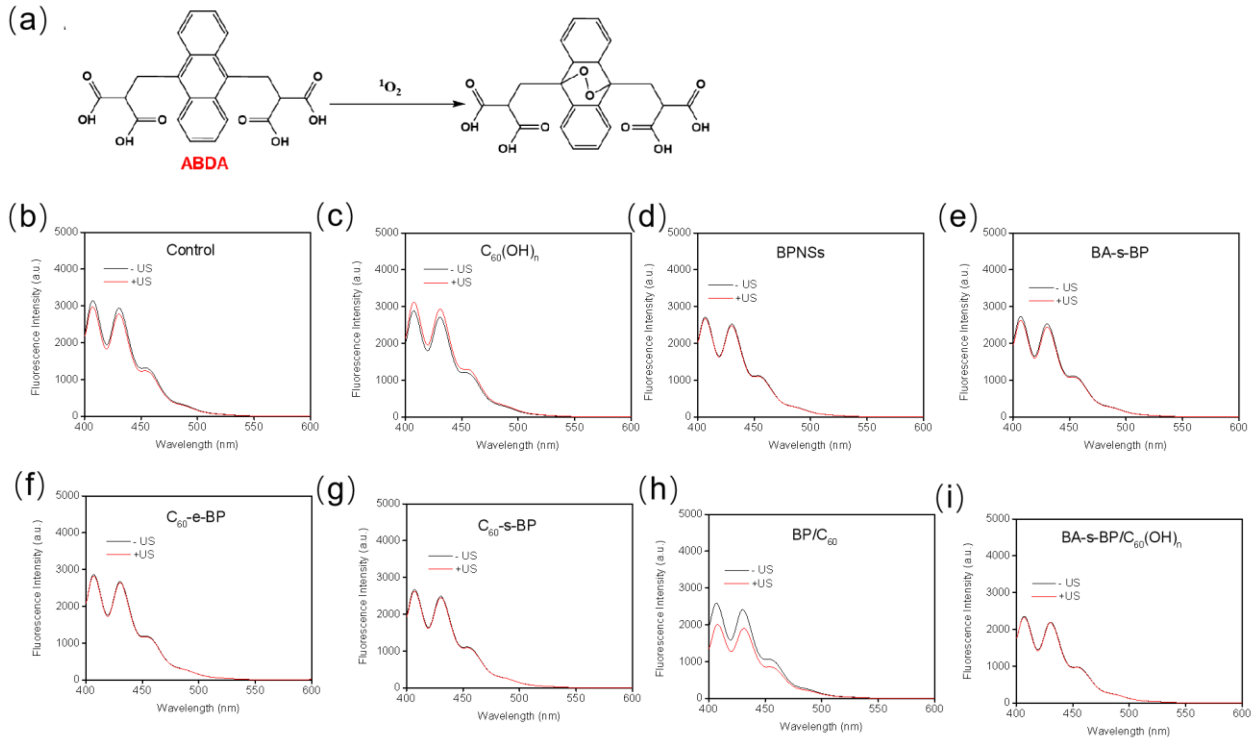

Figure S15. (a) Reaction of ABDA with $^1O_2$ radicals. (b-i) Fluorescence emission spectra obtained by using ABDA as the $^1O_2$ probe.

S18. Reaction of DHE with ·O_2^- radicals and fluorescence emission spectra obtained by using DHE as the ·O_2^- probe.

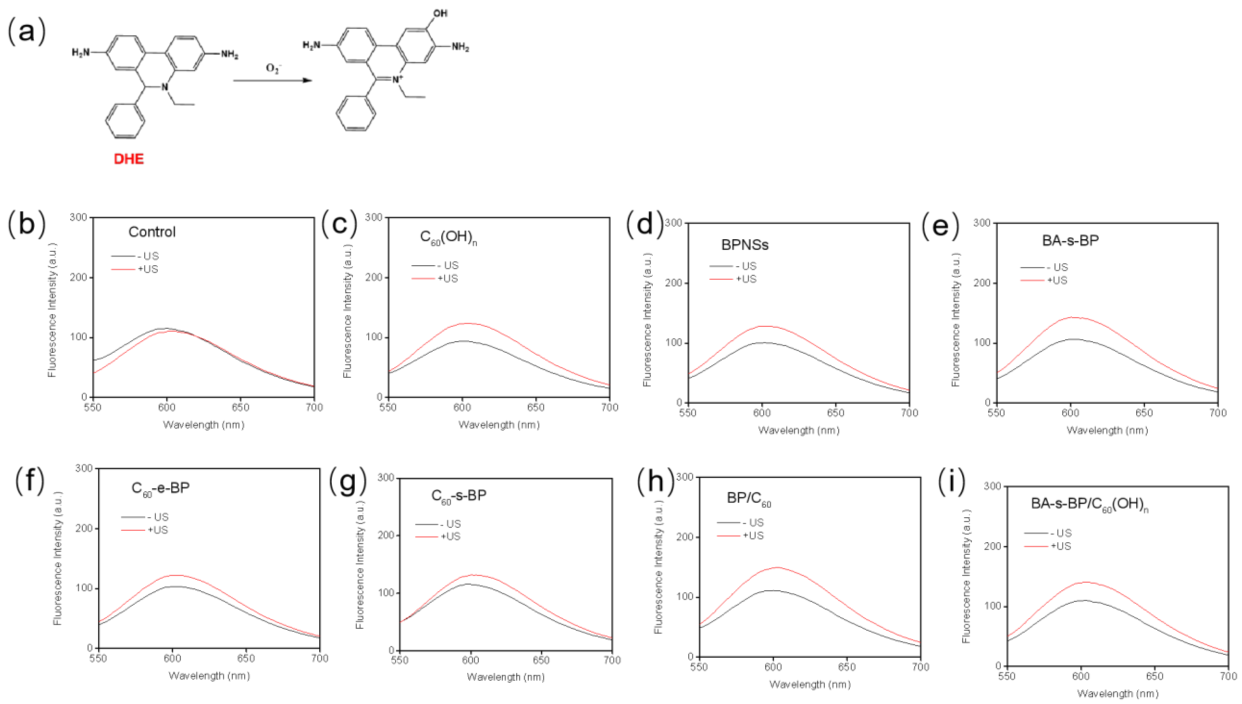

Figure S16. (a) Reaction of DHE with ·O^{2-} radicals. (b-i) Fluorescence emission spectra obtained by using DHE as the ·O^{2-} probe.

S19. In vitro cytoxicity of BP to different cells reported in the literature

Table S3. In vitro cytoxicity of BP to different cells reported in the literature.

BP	Average thickness (nm)	Lateral size (nm)	BP concentrations (μg/mL)	Cell line	Cell viability (%)		Ref
					24h	48h	
BP-1	91.9 ± 32.0	884.0 ± 102.2	50	NIH 3T3	0	0	3
				HCoEpiC	53	6.7	
				293T cells	0	0	
BP-2	27.0 ± 12.0	425.5 ± 78.8	50	NIH 3T3	6.1	0	
				HCoEpiC	78.6	21.2	
				293T cells	0	0	
BP-3	17.4 ± 9.1	208.5 ± 46.9	50	NIH 3T3	46.8	6.1	
				HCoEpiC	118.2	88.1	
				293T cells	74.2	56.9	
BP-4	~10	200	16	hMSCs	96.9	83	4
				QSG-7710	86.9	66.4	
				A549	56.2	23.9	
				Hela	49.1	14.4	
				MCF-7	34.9	10	
BP-5	4~5	150	100	HEK293	87.7	77.9	5
				A549	63.9	36.9	
				D551	105.8	93.4	
				Hela	97.1	102.3	

S20. In vitro cytotoxicity of different samples against 4T1, Hela and NIH-3T3 cells at different incubation times.

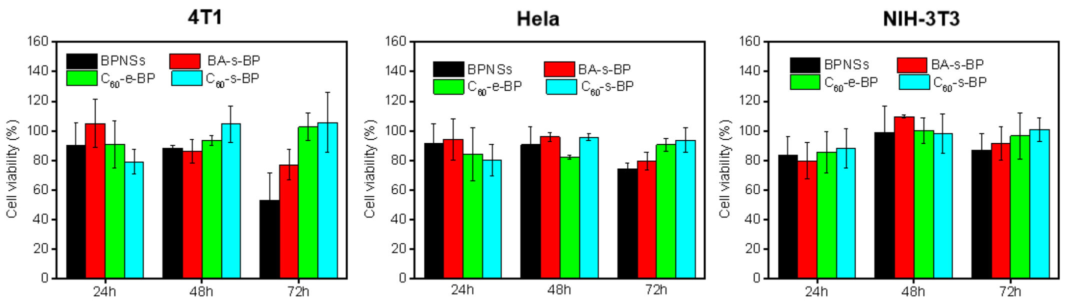

Figure S17. In vitro cytotoxicity of different samples against 4T1, Hela and NIH-3T3 cells at different incubation times.

S21. Detecting the intracellular ROS with DCFH-DA.

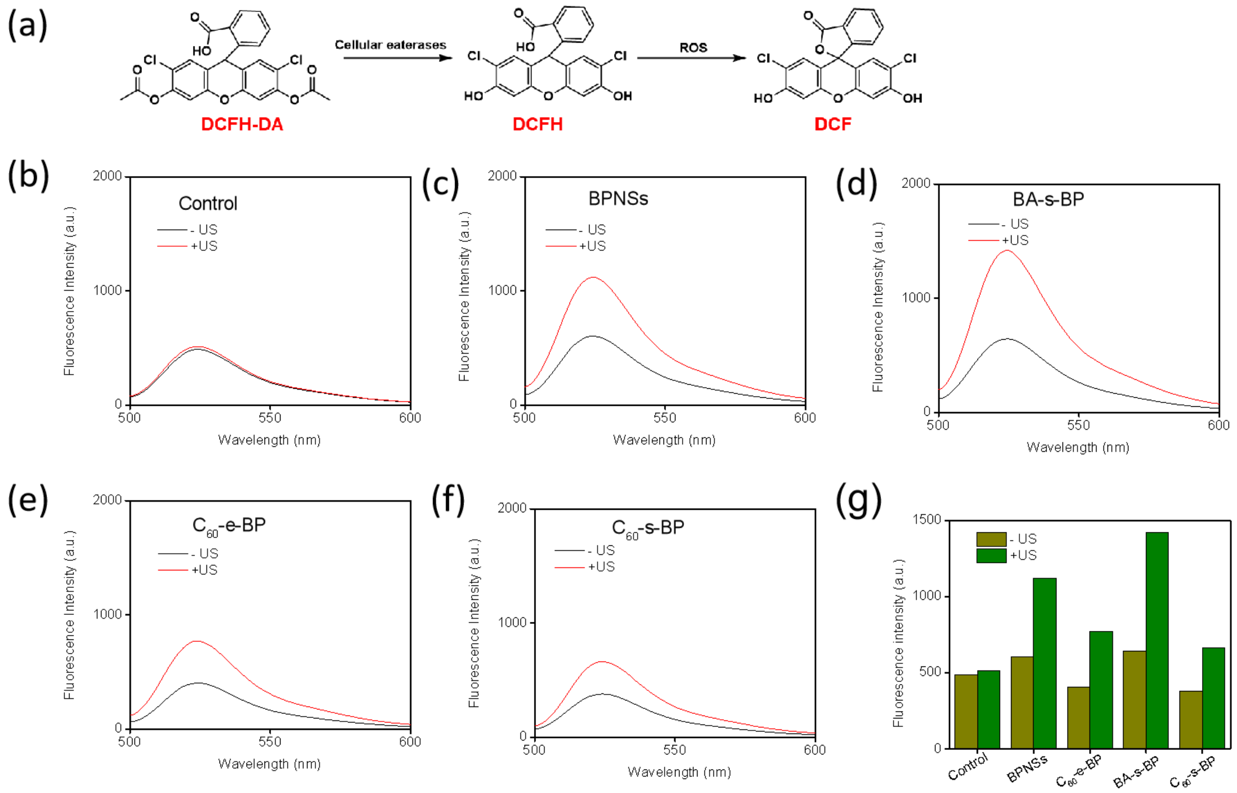

Figure S18. (a) Mechanism of DCFH-DA detecting the intracellular ROS. (b-f) Fluorescence emission spectra obtained by using DCFH-DA as the probe for detecting intracellular ROS generated in 4T1 cells. (g) Intracellular fluorescence emission intensity of DCF in BP or different functional groups modified BP treated 4T1 cells with or without ultrasound irradiation.

S22. The average mouse body weights through the observation window.

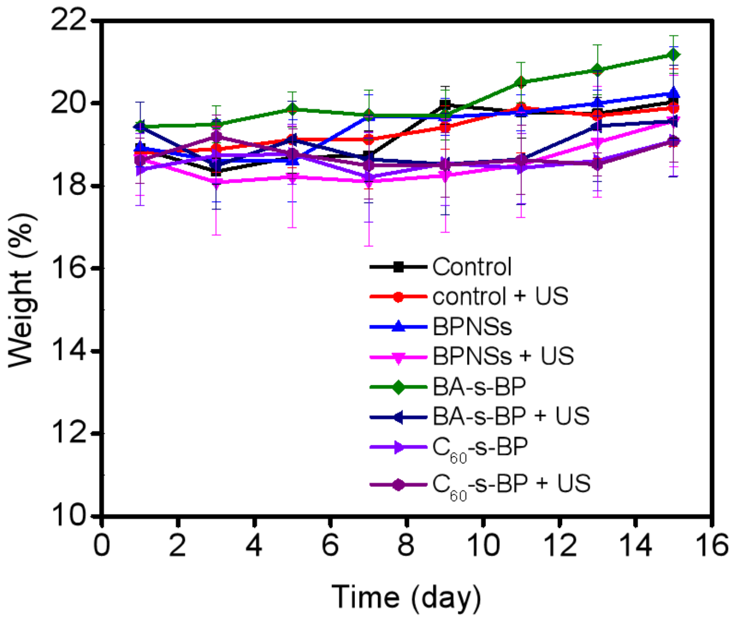

Figure S19. The average mouse body weights through the observation window.

S23. The H&E stained tissue slices of five main organs (Heart, liver, spleen, lung, Kidney) collected on day 16 of treatment.

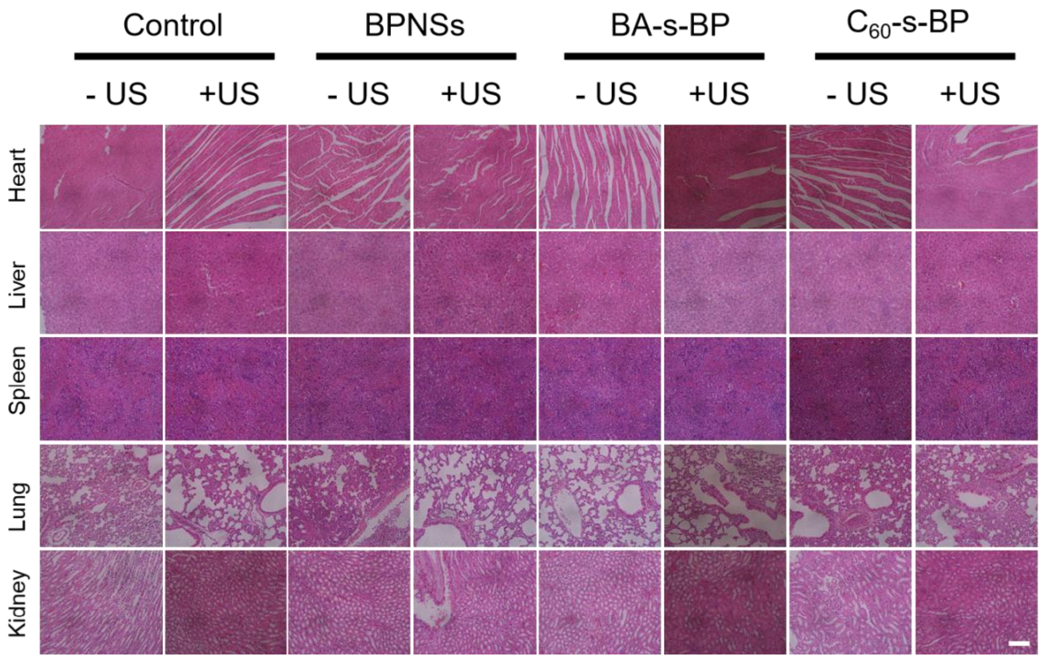

Figure S20. The H&E stained tissue slices of five main organs (Heart, liver, spleen, lung, Kidney) collected on day 16 of treatment. Scale bar=100 µm

S24. Energy level diagrams of the pristine and functionalized BPNSs.

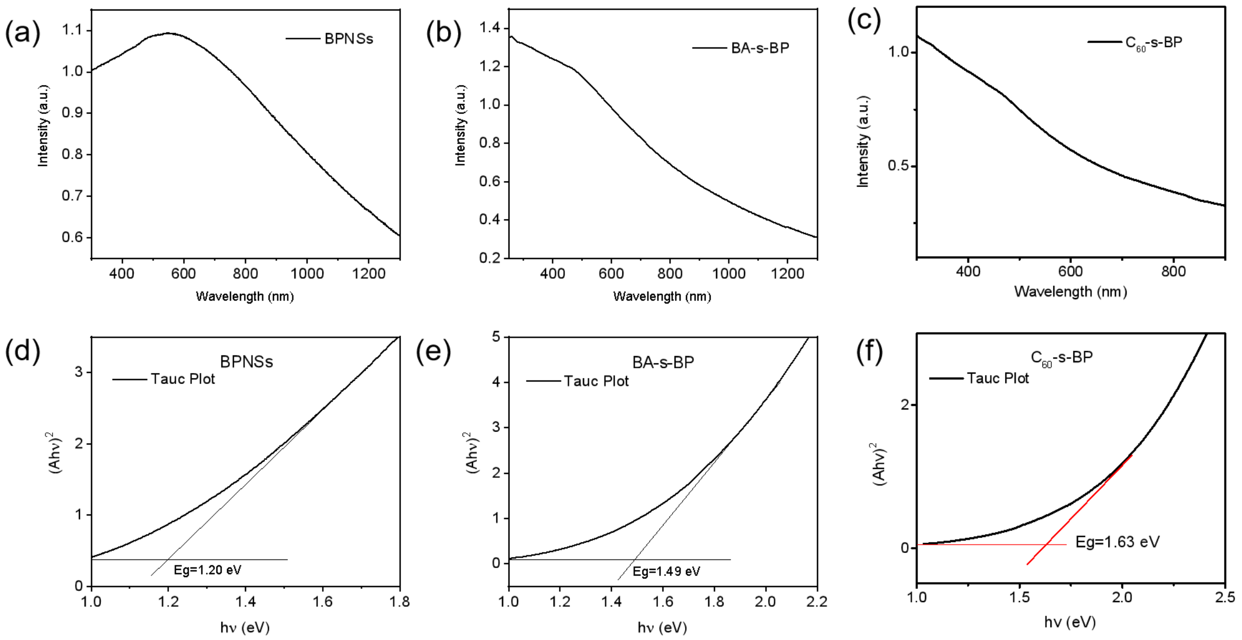

Figure S21. UV-vis absorption spectra of the pristine BPNSs (a), BA-s-BP (b), C_{60}-s-BP hybrid (c). $(ahv)^2$ versus hv curve of the pristine BPNSs (d), BA-s-BP (e) and C_{60}-s-BP hybrid (f). BP is a direct bandgap materials, thus r=2 for Tauc plot $(ahv)^r$ versus hv.

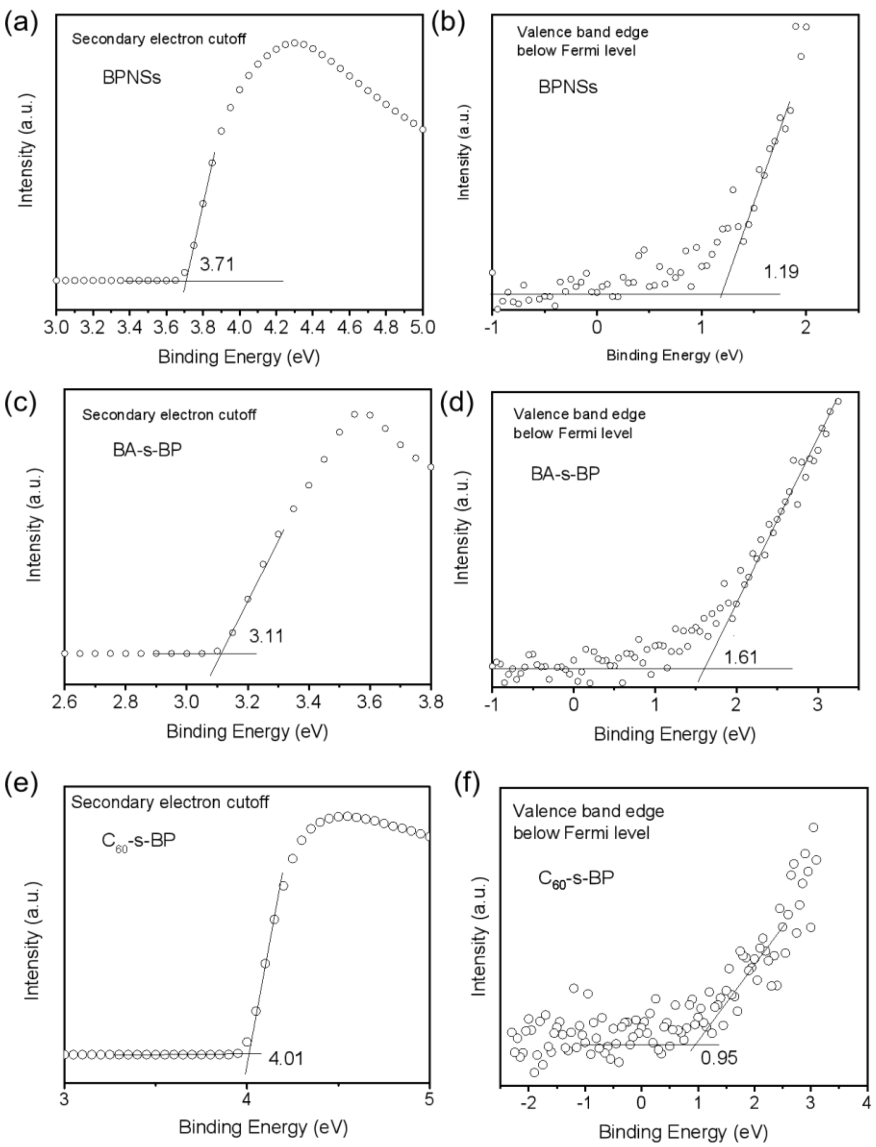

Figure S22. Synchrotron radiation photoemission spectra (SR-PES) of BPNSs (a,b), BA-s-BP (c,d), C_{60}-s-BP hybrid (e, f). The left and right panels show the valance band edges and secondary electron cutoffs, respectively.

Table S4. Fermi level (E_F), CB, VB energy levels and bandgaps of the pristine and functionalized BPNSs.

Sample	Bandgap (eV)	Fermi Level (E_F, vs vacuum level)	Fermi Level (E_F, vs RHE)	Valence band (E_{VB}, vs RHE)	Conduction band (E_{CB}, vs RHE)
BPNSs	1.20	-3.71	-0.79	0.40	-0.80
BA-s-BP	1.49	-3.11	-1.39	0.22	-1.27
C_{60}-s-BP	1.63	-4.01	-0.49	0.46	-1.17
C_{60}-e-BP	1.37	-4.18	-0.32	0.14	-1.23

Synchrotron radiation photoemission spectra (SR-PES) was carried out to determine the energy band structure of the BPNSs, BA-s-BP and C_{60}-s-BP hybrid. The work function (W_F) is determined from the secondary electron threshold as W_F=h*v*-E_{th}, where hv is the photon energy (40 eV). Eth is the secondary electron threshold energy.

According to conversion of energy level of vacuum (-4.5 eV) to energy level vs RHE (0.0 V), E_F (vs RHE) = -4.5-E_F (vs vacuum level).[2]

E_{VB}=|the value below fermi level| + E_F

E_{CB}= Bandgap - |E_{VB}|

E_{VB} is determined from valence band edge determined by SR-PES spectra in Supplementary Figure S22b, d, f.

Bandgap is estimated from $(ahv)^2$ versus hv curve shown in Supplementery Figure S21d, e, f.

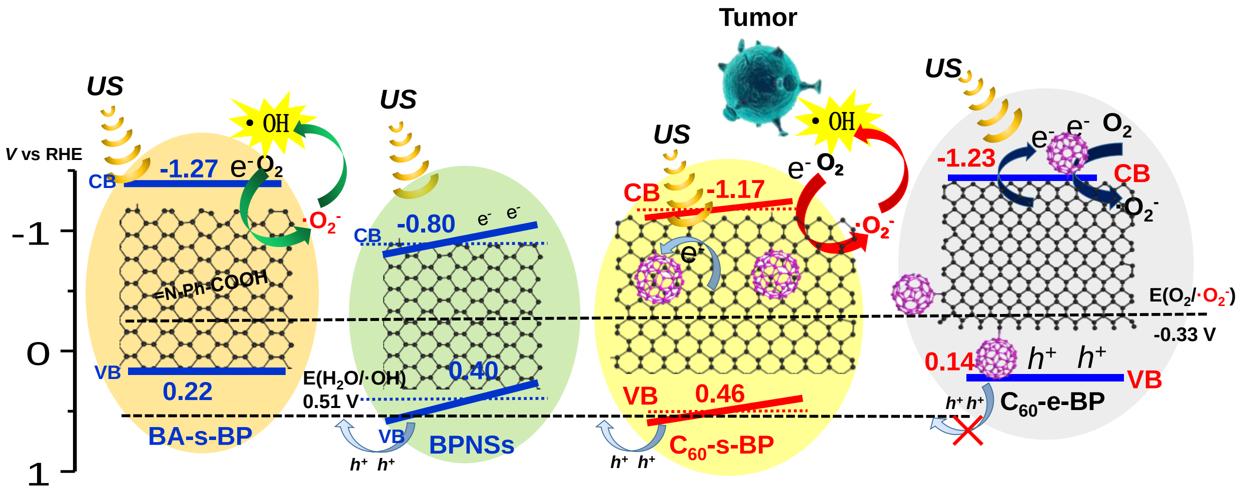

Figure S23. Energy level diagrams of the pristine and functionalized BPNSs.

References

[1] Y. J. Liu, P. F. Gao, T. M. Zhang, X. J. Zhu, M. M. Zhang, M. Q. Chen, P. W. Du, G.-W. Wang, H. X. Ji, J. L. Yang, S. F. Yang, *Angew. Chem. Int. Ed.* **2019**, *58*, 1479.

[2] X. J. Zhu, T. M. Zhang, D. C. Jiang, H. L. Duan, Z. J. Sun, M. M. Zhang, H. C. Jin, R. N. Guan, Y. J. Liu, M. Q. Chen, H. X. Ji, P. W. Du, W. S. Yan, S. Q. Wei, Y. L. Lu, S. F. Yang, *Nat. Commun.* **2018**, *9*, 4177.

[3] X. J. Zhang, Z. M. Zhang, S. Y. Zhang, D. Y. Li, W. Ma, C. X. Ma, F. C. Wu, Q. Zhao, Q. F. Yan, B. S. Xing, *Small* **2017**, *13*, 1701210.

[4] W. H. Zhou, T. Pan, H. D. Cui, Z. Zhao, P. K. Chu, X.-F. Yu. *Angew. Chem. Int. Ed.* **2019**, *58*, 769.

[5] N. Kong, X. Y. Ji, J. Q. Wang, X. N. Sun, G. Q. Chen, T. J. Fan, W. Y. Liang, H. Zhang, A. Y. Xie, O. C. Farokhzad, W. Tao, *Nano. Lett.* **2020**, *20*, 3943.
